# Genomic comparison of sporeforming bacilli isolated from milk

**DOI:** 10.1186/1471-2164-15-26

**Published:** 2014-01-14

**Authors:** Andrea I Moreno Switt, Alexis D Andrus, Matthew L Ranieri, Renato H Orsi, Reid Ivy, Henk C den Bakker, Nicole H Martin, Martin Wiedmann, Kathryn J Boor

**Affiliations:** 1 345 Stocking Hall, Department of Food Science, Cornell University, Ithaca, NY 14853, USA

## Abstract

**Background:**

Sporeformers in the order Bacillales are important contributors to spoilage of pasteurized milk. While only a few *Bacillus* and *Viridibacillus* strains can grow in milk at 6°C, the majority of *Paenibacillus* isolated from pasteurized fluid milk can grow under these conditions. To gain a better understanding of genomic features of these important spoilage organisms and to identify candidate genomic features that may facilitate cold growth in milk, we performed a comparative genomic analysis of selected dairy associated sporeformers representing isolates that can and cannot grow in milk at 6°C.

**Results:**

The genomes for seven *Paenibacillus* spp., two *Bacillus* spp., and one *Viridibacillus* sp. isolates were sequenced. Across the genomes sequenced, we identified numerous genes encoding antimicrobial resistance mechanisms, bacteriocins, and pathways for synthesis of non-ribosomal peptide antibiotics. Phylogenetic analysis placed genomes representing *Bacillus*, *Paenibacillus* and *Viridibacillus* into three distinct well supported clades and further classified the *Paenibacillus* strains characterized here into three distinct clades, including (i) clade I, which contains one strain able to grow at 6°C in skim milk broth and one strain not able to grow under these conditions, (ii) clade II, which contains three strains able to grow at 6°C in skim milk broth, and (iii) clade III, which contains two strains unable to grow under these conditions. While all *Paenibacillus* genomes were found to include multiple copies of genes encoding β-galactosidases, clade II strains showed significantly higher numbers of genes encoding these enzymes as compared to clade III strains. Genome comparison of strains able to grow at 6°C and strains unable to grow at this temperature identified numerous genes encoding features that might facilitate the growth of *Paenibacillus* in milk at 6°C, including peptidases with cold-adapted features (flexibility and disorder regions in the protein structure) and cold-adaptation related proteins (DEAD-box helicases, chaperone DnaJ).

**Conclusions:**

Through a comparative genomics approach we identified a number of genomic features that may relate to the ability of selected *Paenibacillus* strains to cause spoilage of refrigerated fluid milk. With additional experimental evidence, these data will facilitate identification of targets to detect and control Gram positive spore formers in fluid milk.

## Background

Microbial spoilage of foods, particularly highly perishable foods (such as fluid milk produced by high temperature short time [HTST] pasteurization), is a considerable challenge that needs to be addressed to successfully supply food for an anticipated world population of 9 billion people by 2050 [1]. For example, while approximately 87 billion liters of milk are produced in the US annually [2,3], as much as 23 billion liters is potentially lost every year due to microbial spoilage [4]. Whereas manufacturing-related spoilage (e.g. post processing contamination) can be eliminated, contamination with Gram-positive sporeformers, which are microorganisms that can survive pasteurization, is not easily eliminated [5-8]. Among these sporeformers, those that can grow at refrigeration temperatures are a specific concern with regard to food spoilage and spoilage of fluid milk in particular. Two bacterial genera, *Bacillus* and *Paenibacillus,* have been found to be the predominant aerobic sporeformers isolated from pasteurized milk [5,7-11]. *Bacillus* spp. and *Paenibacillus* spp. have also been isolated from dairy farm environments and processing facilities [12], suggesting that these spoilage organisms can be introduced at various stages of the dairy production chain. While the predominant sporeformers isolated early in the shelf life of HTST pasteurized milk are typically *Bacillus* spp., later in the shelf life a shift in bacterial ecology has been reported with *Paenibacillus* spp. representing the majority of sporeformers isolated [7,8], suggesting that *Paenibacillus* spp. may, on a population basis, show a better ability to grow in milk stored at refrigeration temperatures. Molecular and phenotypic characterization of >1,200 sporeformers isolated from milk production not only supported that *Bacillus* spp. and *Paenibacillus* spp. represented the phylogenetic groups most common among aerobic sporeformers isolated from milk, but also identified a considerable number of *Viridibacillus* spp. isolates [5]. This study also showed that while most *Paenibacillus* spp. display β-galactosidase activity and were able to grow in milk at 6°C, only few *Bacillus* spp. and *Viridibacillus* spp. isolates show these phenotypic characteristics. It has been well established though that isolates representing specific *Bacillus* spp. (*B. weihenstephanensis*) and *Viridibacillus* spp. (*V. arenosi*) typically have the ability to grow in refrigerated milk [5]. While *Paenibacillus* spp. are thus the main aerobic sporeformer group of concern as a spoilage organism for the fluid milk industry, some *Bacillus* and *Viridibacillus* spp. may also cause spoilage problems in these types of products.

Considerable efforts have been undertaken to analyze and characterize the genomes of *Bacillus cereus* sensu lato strains, which include human pathogenic species (e.g. *B. anthracis*[13,14]), and foodborne pathogens (e.g. *B. cereus*[14,15]). Conversely, our current knowledge of *Paenibacillus* spp. is rather limited. Recently, a number of *Paenibacillus* genomes, representing strains isolated from different environments (e.g. soil, honeybees, fermented food), have been sequenced and their genomes have been released ([16-25]); however, only a few studies have reported genomic characterization and phylogeny of the sequenced genomes [26-31]. Species belonging to the *Paenibacillus* genus have been isolated from diverse environments including soil, water, milk, insect larvae, and humans [30,32]. Some genomic characteristics associated with the adaptation and survival of *Paenibacillus* in different environments include genes encoding nitrogen fixation enzymes, or genes encoding antimicrobial compounds and bacteriocin production [29,32]. To date, genomic characteristics of *Paenibacillus* strains associated with spoilage of fluid milk remain unknown. Here we analyzed the genomes of isolates representing common *rpoB* allelic types of *Bacillus*, *Paenibacillus* and *Viridibacillus* spp. that were found to contaminate pasteurized milk [5]. The aim was to explore genomic characteristics linked with phenotypic characteristics related to milk spoilage, such as the ability to grow in milk at 6°C and proteolysis of milk proteins, such as casein.

## Results and discussion

The genomic comparisons and supporting phenotypic experiments reported here focused on (i) developing an improved understanding of the genomics of sporeforming food spoilage organisms in the order Bacillales and (ii) identifying specific genomic features that may allow specific members of this order to grow in milk at refrigeration temperatures. As all isolates sequenced here represent species and genetic types that are frequently isolated from commercially processed dairy products, we also hypothesized that genomic analyses may identify novel generally recognized as safe (GRAS) compounds and enzymes that are predicted to be produced by these organisms and that may be useful for food applications (e.g. novel bacteriocins). This hypothesis is supported by previous studies that have identified, through non-genomics classical screening approaches, GRAS bacteriocins from *Paenibacillus* isolates [33].

### Phylogenomic analysis places *Bacillus, Paenibacillus* and *Viridibacillus* into three highly divergent clades, including three distinct *Paenibacillus* clades

The genomes of ten sporeforming Gram positive bacteria isolated from pasteurized milk and dairy farm environments (feed mix, see Table 1) were sequenced; these isolates represented *Paenibacillus* spp. (n = 7), *Bacillus* spp. (n = 2) and *Viridibacillus* sp. (n = 1) (Table 1). Genome sequencing was conducted with the Illumina HiSeq; the draft genomes reported here represented 47 contigs for *Viridibacillus* sp., 106 to 122 contigs for *Bacillus* spp., and 66 to 161 contigs for *Paenibacillus* spp. isolates (see Table 1 for detailed genome assembly statistics). Due to the fact that the genomes were not closed, it is possible that specific genes or orthologs could be misclassified as absent in a given genome; this should not affect the overall enrichment analyses reported below though and absence of specific genes was confirmed in selected target genomes through read mapping (see Methods). Genome sizes (as inferred from the total assembly size) for the two *Bacillus* strains sequenced here were approximately 5.6 Mb, while the genome sizes for the *Paenibacillus* spp. isolates ranged from approx. 6.4 to 7.6 Mb (Table 1). The *Viridibacillus* strain sequenced represented the smallest genome (4.4 Mb). These genome sizes are consistent with previous data, which also found smaller genome sizes for *Bacillus* isolates (ranging from 3.7 Mb for *B. pumilus* [GenBank: CP000813] [34] to 5.7 Mb for *B. pseudomycoides* [GenBank: CM000745]) as compared to *Paenibacillus* spp. isolates; previously reported genome sizes for *Paenibacillus* spp. isolates ranged from 5.3 Mb (*P. polymyxa;* GenBank: CP000154 [35]) to 8.6 Mb (*P. mucilaginosus;* GenBank: CP002869 [25]). It has been previously suggested [18] that this difference in genome sizes may indicate a role of widespread horizontal gene transfer or gene duplication in the evolution of different *Paenibacillus* species.

**Table 1:** Strains and genome description of strains used in this study

**Strain**	**Species**^**1**^	***rpo*** **B allelic type (frequency of AT as reported by Ivy et al. (2012))**^**2**^	**Source (day of shelf life)**	**Total assembly size (number of contigs)**^**3**^	**N50**	**Mol% (G + C)**^**4**^	**GenBank accession number**
FSL R5-860	*Bacillus* sp*.*^*5*^	158 (10.6%)	Pasteurized 2% milk (7 days)	5.6 Mb (106)	337,248	35.0	ASPZ00000000
FSL H7-687	*Bacillus weihenstephanensis*	3 (1.5%)	Pasteurized 2% milk (12 days)	5.6 Mb (112)	405,076	35.2	ASPY00000000
FSL R5-213	*Viridibacillus arenosi*	17(1.8%)	Pasteurized 2% milk (7 days)	4.4 Mb (47)	661,909	35.3	ASQA00000000
FSL H8-237	*Paenibacillus odorifer*	15 (9.5%)	Pasteurized 2% milk (21 days)	7.3 Mb (128)	256,380	44.0	ASPV00000000
FSL R7-277	*Paenibacillus* sp*.*^6^	45 (0.2%)	Pasteurized 2% milk (7 days)	7.6 Mb (122)	257,268	52.5	ASPX00000000
FSL R7-269	*Paenibacillus* sp*.*^6^	163 (0.7%)	Pasteurized 2% milk (7 days)	7.5 Mb (161)	252,603	51.8	ASPS00000000
FSL R5-192	*Paenibacillus amylolyticus*	23 (2.7%)	Pasteurized 2% milk (7 days)	7.0 Mb (80)	383,457	45.8	ASPR00000000
FSL H7-689	*Paenibacillus amylolyticus*	23 (2.7%)	Pasteurized 2% milk (12 days)	6.8 Mb (66)	525,446	45.9	ASPU00000000
FSL R5-808	*Paenibacillus glucanolyticus*	159 (0.5%)	Pasteurized 2% milk (1 day)	6.4 Mb (89)	316,677	48.8	ASPT00000000
FSL H8-457	*Paenibacillus lautus*	117 (0.1%)	Feed mix (N/A)	7.0 Mb (69)	696,553	51.2	ASPW00000000

^1^Putative species were inferred based on 16S rRNA phylogenies (using 16S rRNA sequences extracted from the draft genome sequences) and confirmed using phylogenetic analysis of 31 protein sequences (Figure 1).

^2^Percentage of isolates with a given allelic type (AT) from a total of 1,288 isolates tested.

^3^Only contigs >199 bp are included.

^4^Inferred from genome sequence.

^5^FSL R5-860 was designated as *Bacillus* sp. as this strain clusters with *B. cereus* in a 16S rRNA phylogeny, but clusters with *B. weihenstephanensis* in the 31 protein phylogeny (Figure 1); while this is consistent with previous reports that *B. cereus* is polyphyletic, final species assignment of this isolate will require sequencing and analysis of further *Bacillus* isolates.

^6^Isolates FSL R7-277 and R7-269 were classified as *Paenibacillus* sp. as they could not be classified to species based on 16S rRNA sequences; in a 16S rRNA phylogeny both of these isolates clustered with *P. caespitis* (GenBank accession no. AM745263), but were not assigned this species name as this species has not yet been validly published.

**Figure 1:**
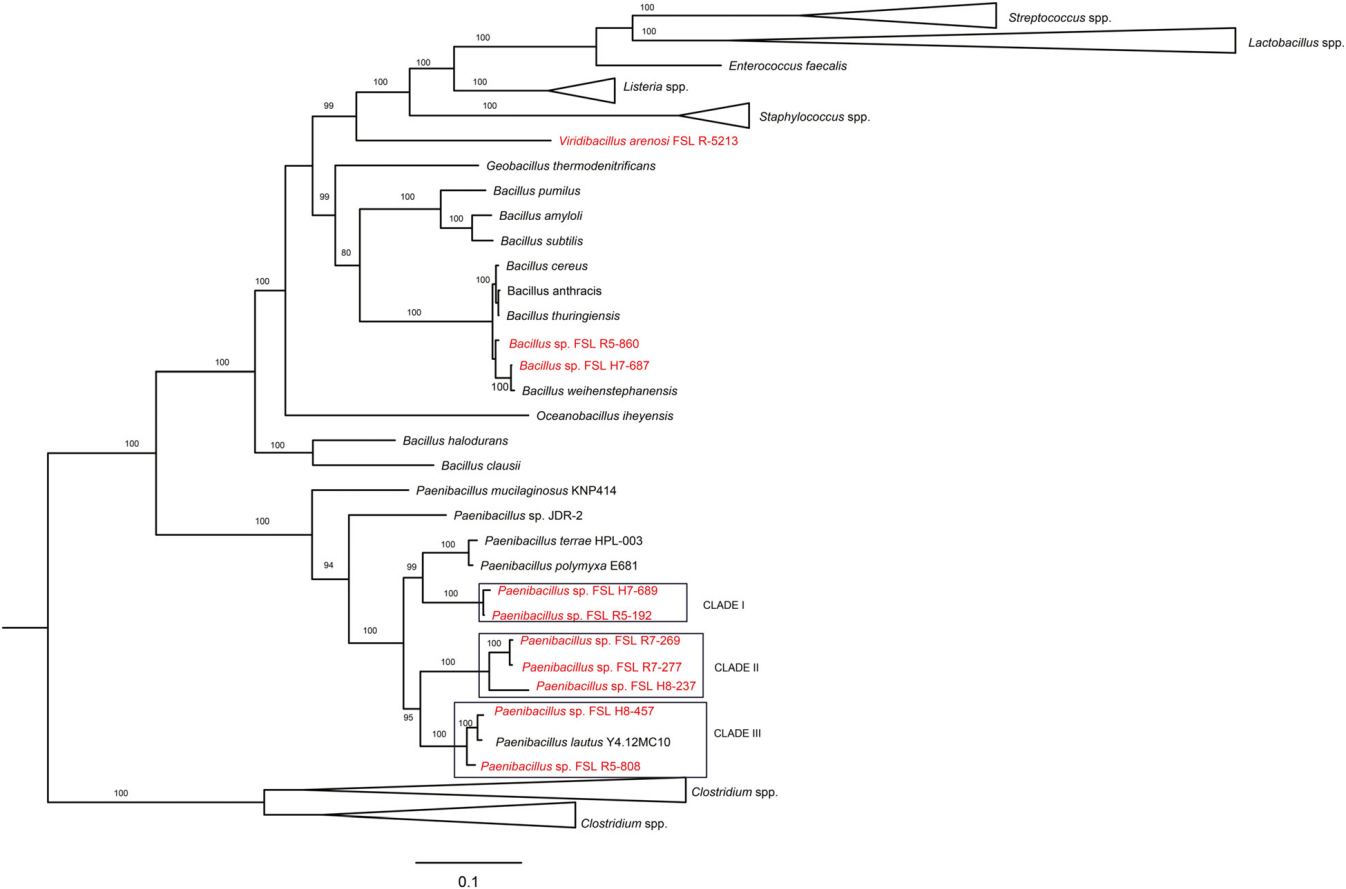
**Maximum likelihood phylogeny from a concatenated amino acid sequence of 31 core genes.** Genomes sequenced here as well as data for selected *Bacillus, Paenibacillus*, and additional Firmicutes were used to obtain the gene sequences for the phylogenetic analysis. The clade containing *Clostridium* spp. was selected to root the tree. For better visualization of the genera of interest (*Bacillus, Paenibacillus,* and *Viridibacillus*), branches that contained genomes of other genera were collapsed. Numbers on the branches represent bootstrap values calculated with 1000 replicates. Bootstrap values <80 are not included. Strains sequenced in this study are shown in red. The three *Paenibacillus* clades are labeled.

Phylogenetic analysis of the genomes sequenced here, along with 52 genomes representing selected previously sequenced Firmicutes, including five additional *Paenibacillus* and nine additional *Bacillus* (Figure 1 and Additional file 1), was performed using amino acid (aa) sequences encoded by 31 core genes previously reported to represent suitable phylogenetic markers for bacteria [36]. This analysis places *Bacillus*, *Paenibacillus* and *Viridibacillus* into three distinct well supported clades, which is consistent with reclassification, in 1993, of species now designated as *Paenibacillus* into a genus distinct from the genus *Bacillus*[37]. As no genome sequence for the genus *Viridibacillus* has previously been reported, the analyses reported here also provided an opportunity to clarify the phylogenetic position of a representative of this genus. Based on the 31 protein sequence phylogeny, the *V. arenosi* strain sequenced here falls into a distinct clade that shares a common ancestor with a number of non-sporeforming Firmicutes (Figure 1). This not only confirms that *Viridibacillus* is distinct from *Bacillus* and *Paenibacillus*, but also suggests a single emergence of the most recent common ancestor of non-sporeforming Firmicutes from a sporeforming ancestor.

Our phylogenetic analysis of 31 protein sequences (Figure 1) also identified three distinct well supported clades among the *Paenibacillus* genomes sequenced here, including (i) clade I, which contains the two *P. amylolyticus* strains (FSL H7-689 and FSL R5-192), (ii) clade II, which contains the two *Paenibacillus* spp. strains (FSL R7-277 and FSL R7-269) and one *P. odorifer* (FSL H8-237) strain, and (iii) clade III, which contains two *P. lautus* strains (one sequenced in here [FSL H8-457] and one previously sequenced [Y4.12MC10]) and one *P. glucanolyticus* strain (FSL R5-808) (Figure 1). These three clades are well supported (100% bootstrap); however, the number of genomes characterized here is small and additional studies will be necessary to further validate the three clades identified here. Considerable genome divergence among the analyzed *Paenibacillus* genomes is also supported by an overall genome alignment (see Additional file 2). Analysis of the average nucleotide identity based on the BLAST algorithm (ANIb) scores for *Paenibacillus* isolates sequenced here confirmed that isolates FSL H7-689 and FSL R5-192 (both classified as *P. amylolyticus* based on 16S rRNA) represent closely related genomes (ANIb = 97.39%, over 92.18% of the genomes; Additional file 3), which also supports their classification into the same species (as an ANI of 95% has been proposed as species cut-off [38]). On the other hand the genomes of strains FSL R7-269 and FSL R7-277 (both classified as *Paenibacillus* spp., according to 16S rRNA (see Table 1)) show an ANIb of 92.54% (over 72.88% of genome; Additional file 3), suggesting that these two strains represent separate species despite a 1223/1225 (> 94.99%) nucleotide 16S rRNA identity. This finding is consistent with previous reports that 16S rRNA sequence divergence statistics show less resolution for bacterial species designation than the ANIb values [38].

### *Paenibacillus* and *Bacillus* genomes carry a diversity of antimicrobial resistance genes

Characterization of phenotypic antimicrobial resistance patterns among the strains sequenced here (Table 2) found resistance to at least one antibiotic in all but two strains. The most common resistance pattern (lincomycin-penicillin) was found in two *Bacillus* and two *Paenibacillus* isolates (Table 2); in addition, one strain showed resistance to lincomycin-streptomycin, two strains showed resistant to streptomycin only, and one strain showed resistance to lincomycin only (Table 2). While this is, to our knowledge, the first report of antimicrobial resistant *Bacillus* and *Paenibacillus* isolates from milk, previous studies have reported antimicrobial resistance in disease associated *Paenibacillus* isolates, including (i) resistance to metronidazole [30], tetracycline [39], and glycopeptides [40] in *P. larvae*, which is associated with disease in honeybees and (ii) multi-drug resistance (to glycopeptide, beta-lactams, aminoglycosides, macrolides and lincosomides) in a *Paenibacillus* isolated from fertile soil in India [41]. Similarly, antimicrobial resistant *Bacillus* isolates have previously been isolated from a variety of clinical [42-44] and non-clinical [45] sources.

**Table 2:** Phenotypic characterization of strains used in this study

**Strain**	**Species**	**Relative growth, at day 21 (6°C)**^**1**^	**Relative proteolysis on Skim Milk Agar (24 h 32°C)**	**β-gal activity**	**Antimicrobial resistance pattern**^**3**^
FSL R5-860	*Bacillus* sp.	-	+	-	Lincomycin, Penicillin
FSL H7-687	*Bacillus weihenstephanensis*	++	+	-	Lincomycin, Penicillin
FSL R5-213	*Viridibacillus arenosi*	++	- ^2^	-	Lincomycin
FSL H8-237	*Paenibacillus odorifer*	++	+	+	Not tested^4^
FSL R7-277	*Paenibacillus* sp.	+	+	+	Streptomycin
FSL R7-269	*Paenibacillus* sp.	+	+	+	Streptomycin
FSL R5-192	*Paenibacillus amylolyticus*	-	+	+	Lincomycin, Streptomycin
FSL H7-689	*Paenibacillus amylolyticus*	++	+	+	Pansusceptible
FSL R5-808	*Paenibacillus glucanolyticus*	-	-	+	Lincomycin, Penicillin
FSL H8-457	*Paenibacillus lautus*	-	-	+	Lincomycin, Penicillin

^1^Relative growth in skim milk broth, plated on BHI at day 21 of shelf life (storaged at 6°C): (−) <1 log cfu/mL, (+) 1–6 log cfu/mL, (++) >6 log cfu/mL.

^2^Isolate produced pinkish coloration of Skim Milk Agar.

^3^Isolates were tested for susceptibility against 16 antimicrobials included in the Gram positive NARMS panel (see Methods).

^4^Strain was considered too fastidious for susceptible testing.

Analyses of the sequenced genomes also identified putative antimicrobial resistance genes, in both pan-susceptible and resistant *Bacillus* and *Paenibacillus* isolates (Additional file 4). Most genomes contained more than 20 putative antimicrobial resistance genes (including genes putatively encoding heavy metal resistance characteristics; see Additional file 4). For example, we identified, in the genomes sequenced here, genes putatively encoding resistance to β-lactams (e.g. metallo-β-lactamases and TEM β-lactamase), aminoglycosides (e.g. aminoglycoside 3’phosphotransferase), chloramphenicol (e.g. chloramphenicol acetyltransferase), copper (e.g. CopC, and PcoD), vancomycin (e.g. VanZ), arsenic (e.g. Acr3), tellurite (e.g. TerD), and quaternary ammonium (e.g. EmrE) as well as genes encoding a number of efflux pumps (e.g. EmrB/QacA, Bcr/CflA). Antibiotic resistance genes have previously been reported in *Paenibacillus* from non-dairy sources [39,46], including reports of resistance genes similar to those found here in *P. vortex* and *P. larvae* genomes [26,47]. Similarly, presence of antimicrobial resistance genes appears to be common in *Bacillus* spp. [42,45,48].

Interestingly, the presence of genes putatively encoding resistance to a given antibiotic did not always correlate to the resistance phenotypes of the respective isolates. For example, while genes encoding β-lactamases were detected in all 10 genomes, only 4 strains showed penicillin resistance (Table 2). Similarly, while the lincomycin resistance operon *lmrAB* was detected in all 10 genomes, only 6 strains showed phenotypic resistance to lincomycin. An alignment of *lmrAB* showed two clades, one that contains only resistant strains and one that contains resistant and susceptible strains (Additional file 5). These observations could be interpreted as being consistent with previous reports that proposed that, in *Paenibacillus* and *Bacillus* isolates, these “antimicrobial resistance genes”, are not necessarily involved in the resistance against anthropogenic antibiotics, but rather encode “defense mechanisms” that allow the bacteria carrying these genes to compete against antagonistic bacteria [26,28]. This hypothesis is also supported by studies that suggest that expression of resistance genes might be tightly regulated in environmental non-pathogenic bacteria and that these genes may not necessarily be expressed under laboratory conditions that are appropriate to evaluate antimicrobial resistance phenotypes in bacterial pathogens. For example, the *Bacillus subtilis* lincomycin resistance operon *lmrAB* has been shown to be dually regulated in response to flavonoids, rather than in response to lincomycin [49].

### A number of bacteriocins and pathways for biosynthesis of non-ribosomal peptide antibiotics are encoded in the genomes of dairy associated *Paenibacillus* and *Bacillus*

*Paenibacillus* spp. have previously been reported to produce certain antimicrobials, like bacteriocins and non-ribosomal peptides antibiotics (NRPA) [18,50-52]. Here we investigated the presence of genes encoding bacteriocins and NRPA biosynthesis in the sequenced isolates, which could represent potential source for GRAS antimicrobials. Using HMM searches, we identified genes encoding putative class II bacteriocins in four of the genomes, including one *Bacillus* strain (FSL H7-687) and three *Paenibacillus* strains (FSL H8-237, FSL R5-192, and FSL H7-689) (Table 3). All of these bacteriocin genes were found in operons that encoded both the bacteriocins and the putative corresponding bacteriocin transporters [53,54]. In the genome of *B. weihenstephanensis* FSL H7-687 we specifically identified a bacteriocin operon that was annotated as encoding a bacteriocin cerein 7B precursor, an ABC-type bacteriocin exporter, and a bacteriocin secretion accessory protein. These proteins present an average of 87% of amino acid (aa) identity to the same operon in *B. cereus* (Table 3). In two of the *Paenibacillus* genomes (FSL H7-689 and FSL R5-192) we identified an operon annotated as encoding the lantibiotic mersacidin biosynthesis protein LanM and the corresponding transporter protein (Table 3). These proteins showed an average aa identity (across both proteins) of 38% (FSL R5-192) and 40% (FSL H7-689) with the corresponding *B. cereus* proteins. In addition, we identified, in the genome of *Paenibacillus* FSL H8-237, another class II bacteriocin operon that was annotated as encoding a bacteriocin with a double-glycine leader peptide, a secretory protein, and an ABC-type bacteriocin transporter. The proteins encoded in this operon present 34% aa identity (across all 3 proteins) with a similar system in *Anoxybacillus flavithermus*. To search for unusual or un-annotated bacteriocins we conducted a search with BAGEL3 [55]. This analysis identified additional putative bacteriocins of class I (i.e., lanthipeptide, lassopeptide, and sactipeptide bacteriocins) in four genomes (FSL H7-687, FSL R7-277, FSL R7-269, and FSL R5-192), as well as two class II bacteriocins (i.e., UvB and un-classified class II bacteriocin) in two genomes (FSL R5-860 and FSL H8-237) (Table 3). While some of the bacteriocins identified show a high level of homology with previously identified bacteriocins, most of the bacteriocins identified in the *Paenibacillus* genomes show low level homology to previously identified bacteriocins, indicating that they may represent novel bacteriocins.

**Table 3:** Antimicrobial systems detected using HMM searches against the sequenced genomes

**Strain (FSL)**^**1**^	**Species**	**Antimicrobial system**^**3**^	**Locus tag**	**pBLAST identity in %**^**6 **^**[species = GenBank acc. number]**
Bacteriocins^2^				
R5-860	*B.* sp*.*	Bacteriocin UvB^4^	C175_07686 to C175_07731	100% [*B. thuringiensis* = WP_003302295]
H7-687	*B. weihenstephanensis*	Bacteriocin cerein 7B	C174_01754 to C174_01764	87.0% [*B. cereus* = CAJ32354.1]
		Lanthipeptide_class_I^4^	C174_05773 to C174_05843	100% [*B. cereus* = WP_002128181.1]
		Lasso_peptide^4^	C174_07587 to C174_07617	100% [*B. cereus* = WP_002128586.1]
R5-213	*V. arenosi*	-	-	-
H8-237	*P. odorifer*	Putative bacteriocins with double-glycine leader peptide	C17_06947 to C171_06937	34.3% [*A. flavithermus* = YP_002316950.1]
		Class II^4^	C171_31256	82% [*Paenibacillus* sp. = WP_018755215.1]
R7-277	*P.* sp*.*	Lasso_peptide^4^	C173_26127 to C173_26182	78% [*Paenibacillus* sp. = WP_019913509.1]
		Sactipeptides^4^	C173_26762 to C173_26742	93% [*Paenibacillus* sp. = WP_017690652.1]
R7-269	*P.* sp*.*	Lasso_peptide^4^	C162_11816 to C162_11871	78% [*Paenibacillus* sp. = WP_019913509.1]
		Sactipeptides^4^	C162_32049 to C162_32089	48% [*Dorea* sp. = WP_005337554.1]
R5-192	*P. amylolyticus*	Lantibiotic mersacidin	C161_23334 to C161_23344	38.0% [*B. cereus* = ZP_17590371]
		Lasso_peptide^4^	C161_09323 to C161_09363	56% [*Paenibacillus* sp. = YP_003009997.1]
		Sactipeptides^4^	C161_03994 to C161_03969	49% [*Paenibacillus* sp. = WP_016818495.1]
H7-689	*P. amylolyticus*	Lantibiotic mersacidin	C170_27843 to C170_27833	40.0% [*B. cereus* = ZP_17590371]
R5-808	*P. glucanolyticus*	-	-	-
H8-457	*P. lautus*	-	-	-
Non ribosomal peptide antibiotics				
R5-860	*B.* sp.	uncharacterized antibiotic^5^	C175_22452 to C175_22455	99.8% [*B. cereus* = NP_832072.1]
		Gramicidin	C175_21698 to C175_21708	98.0% [*B. cereus* = ZP_17623965.1]
H7-687	*B. weihenstephanensis*	Uncharacterized antibiotic^5^	C174_ 09897 to C174_09917	99.0% [*B. weihenstephanensis* = YP_001645029]
		Gramicidin	C174_03013 to C174_03023	98.0% [*B. cereus* = ZP_17640177.1]
R5-213	*V. arenosi*	-	-	-
H8-237	*P. odorifer*	-	-	-
R7-277	*P.* sp*.*	Mycosubtilin	C173_20341 to C173_20411	33.0% [*B. laterosporus* = ZP_16304742.1]
		Cereulide	C173_11825 to C173_11870	32.3% [*N. punctiforme* = YP_001866562.1]
R7-269	*P.* sp*.*	Uncharacterized antibiotic^5^	C162_12938 to C163_12948	37.3% [*N. punctiforme* = YP_001866562.1]
		Gramicidin	C162_20656 to C162_20696	40.8% [*A. cellulolyticus* = ZP_09464526]
		Fusaricidin	C162_28814 to C162_28839	62.5% [*B. thuringiensis* = ZP_04087647.1]
R5-192	*P. amylolyticus*	Gramicidin/polymyxin	C161_02905 to C161_02925	87.2% [*P. polymyxa* = YP_003872502.1]
		Mycosubtilin	C161_22119 to C161_22129	53.5% [*B. atrophaeus* = YP_003973351.1]
		Iturin	C161_04861 to C161_04866	34.0% [*P. polymyxa* = YP_005960904.1]
H7-689	*P. amylolyticus*	Gramicidin	C170_23095 to C170_23105	36.3% [*P. curdlanolyticus* = ZP_07387013.1]
		Gramicidin/polymyxin	C170_04453 to C170_04473	90.2% [*P. polymyxa* = YP_003872502.1]
		Mycosubtilin	C170_20065 to C170_20070	53.5% [*B. atrophaeus* = YP_003973351.1]
		Iturin	C170_00119 to C170_00129	38.0% [*P. polymyxa* = YP_005960904.1]
		Gramicidin S	C170_21789	91.0% [*P. polymyxa* = AEZ51516]
R5-808	*P. glucanolyticus*	Gramicidin	C169_08418 to C169_08438	77.2% [*P. vortex* = ZP_07900219.1]
H8-457	*P. lautus*	-	-	-

^1^The full strain designation includes the prefix FSL, e.g., FSL R5-860.

^2^Bacteriocin genes identified are predicted to encode class I and II bacteriocins.

^3^The designation assigned to a given putative antimicrobial system represents the designation associated with the previously annotated system that showed the highest similarity to the query sequence, or the designation predicted by Bagel3.

^4^Identified by BAGEL3.

^5^The designation “uncharacterized antibiotic” was given to ORFs that contain domains involved in peptide antibiotic synthesis.

^6^Average protein BLAST identity for all proteins encoded in a given operon.

Genes encoding putative NRPA biosynthesis pathways were identified in the two *Bacillus* and five *Paenibacillus* strains (Table 3). In each of the two *Bacillus* strains, we identified two highly similar operons, one annotated as encoding proteins involved in gramicidin biosynthesis and one annotated as encoding proteins involved in biosynthesis of a putative, uncharacterized, antibiotic. The proteins encoded by these operons showed aa identities of >97% (across all proteins in a given operon) to the corresponding proteins in *B. cereus* (Table 3). Similarly, a number of different operons encoding proteins involved in the synthesis of NRPA were detected in five *Paenibacillus* genomes; *Paenibacillus* strains FSL R7-277, FSL R7-269, FSL R5-192, FSL R5-808, and FSL H7-689 were annotated as encoding two, three, three, one, and five NRPA biosynthesis operons, respectively. While NRPA have previously been identified in several *Paenibacillus* spp. [28,56,57], *Paenibacillus* strains without NRPA have also been described [29]. The operons identified here were annotated as encoding proteins required for the biosynthesis of NRPA similar to Mycosubtilin, Musaricidin, Gramicidin S, Cereulide, Iturin, Bacitracin, and Lichenysin (Table 3). Interestingly, many of the proteins encoding these putative NRPA biosynthesis pathways showed low levels of protein identity (e.g. 32%; see Table 3) to corresponding proteins in *Bacillus* genomes. This suggests that many of these operons may encode proteins that facilitate the biosynthesis of novel NRPA, which could potentially represent novel antimicrobials that inhibit dairy associated bacteria. While it is possible that these antimicrobials would be granted GRAS status (as there is evidence for consumption of these spoilage organisms by a considerable number of consumers), additional scientific safety studies may be needed, particularly if efficacy against relevant target organisms is shown.

### Both *Paenibacillus* and *Bacillus* genomes encode proteolytic systems with a putative role in casein breakdown

Phenotypically, both *Bacillus* isolates sequenced here as well as five of the seven *Paenibacillus* isolates showed proteolysis (on skim milk agar at 32°C). BLAST searches of all 10 genomes sequenced here were thus performed to screen for the presence of genes encoding proteolytic systems and proteins previously reported as facilitating casein breakdown in lactic acid bacteria [58]. Using protein sequences for appropriate cell-wall proteinases (Prt), peptide transporters (e.g. oligo- di-tri-peptide transporters), and 13 different peptidases (e.g. PepP, PepA, PepE) [59] as query sequences, we identified cell-wall proteinases as well as oligo-peptide (OppABCD) and di-peptide (DppABCD) transporters in all 10 genomes (Additional file 6). Genes encoding Tri-peptide transporters (DtpT) were found in four genomes (FSL H8-237, FSL H7-687, FSL R5-860, and FSL R5-213), including one strain that did not show proteolytic activity in the phenotypic assay (strain FSL R5-213, see Table 2 and Additional file 6). We also identified genes encoding peptidases in all 10 genomes; the number of peptidase encoding genes ranged from 15 (in FSL R7-269) to 22 (in FSL H7-687 and FSL R5-860) peptidase genes per genome; genes encoding PepD, PepO and PepE/PepC, which had previously been shown to contribute to growth in milk in lactic acid bacteria [60], were not found in any of the 10 genomes (Additional file 6). Overall, even though some of the 10 strains characterized here were negative for proteolysis on skim milk agar, all strains appear to encode proteins for all major steps required for casein breakdown in lactic acid bacteria (i.e., cell-wall proteinase activity, peptide transport and intracellular peptidases). Future experimental work will thus be required to dissect the function of the different putative casein hydrolysis-related proteins encoded in the genomes sequenced here.

### *Paenibacillus* genomes carry several genes encoding β-galactosidase activity

**Figure 2:**
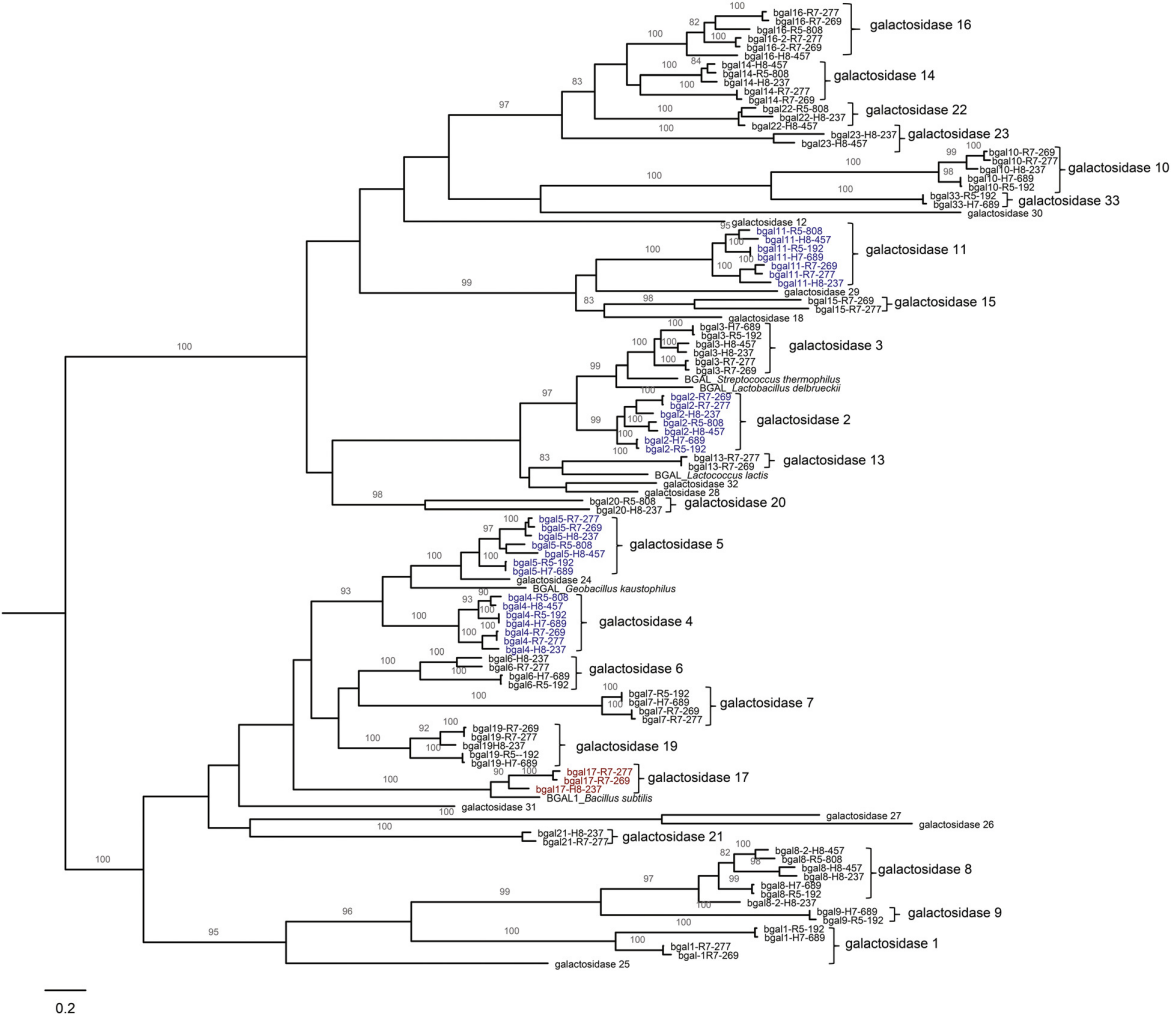
**Maximum likelihood phylogeny of the β-galactosidases identified in the *Paenibacillus *genomes.** β-galactosidases present in all seven *Paenibacillus* genomes are show in blue; the β-galactosidase only present in clade II strains is shown in red. Numbers on the branches represent bootstrap values based on 100 replicates. Bootstrap values <80 are not included. Five previously characterized β-galactosidases were included in the analysis, including LacZ of *Lactococcus lactis* (uniprot: Q48727), *Lactobacillus delbrueckii* (uniprot: P0C1Y0), and *Streptococcus thermophiles* (uniprot: P23989), YesZ of *Bacillus subtilis* (uniprot: O31529), and BgaB of *Geobacillus kaustophilus* (uniprot: P19668).

Consistent with previous phenotypic studies [5], we found that only the seven *Paenibacillus* isolates expressed β-galactosidase activity; the two *Bacillus* and the *Viridibacillus* isolates characterized here were β-galactosidase negative (Table 2). An HMM search for β-galactosidase domains also identified genes with these types of domains only in the seven *Paenibacillus* genomes, but not in the *Bacillus* and *Viridibacillus* genomes (Additional file 7). Interestingly, we identified between 9 and 19 genes encoding putative β-galactosidases in each *Paenibacillus* genome (Additional file 7). A maximum likelihood phylogeny of the aa sequences for all β-galactosidases (Figure 2) not only revealed considerable diversity among these proteins, but also allowed us to classify these proteins into 33 β-galactosidase types (β-gal 1 to 33); putative β-galactosidases were assigned the same type if they clustered into the same clade (see Figure 2). While five β-gal types (2, 4, 5, and 11) were found in each of the seven genomes (Figure 2 & Additional file 8); 13 β-gal types (i.e., 1, 6, 7, 10, 12, 13, 15, 17, 18, 19, 21, 29, and 31) were absent in both strains classified into *Paenibacillus* clade III (FSL H8-457, FSL R5-808) and one β-gal types (β-gal17) was only found in clade II strains (Additional file 8). Both *Paenibacillus* clade I strains contained the identical repertoire of β-gal types.

Similarly to our findings, presence of multiple genes encoding β-galactosidases has been previously described for haloarchaeal bacteria [61]. Interestingly, β-galactosidases have also been identified in microorganisms typically found in lactose-free environments [62] and β-galactosidases in these bacteria have been shown to hydrolyze pectin-like plant polysaccharides [62]. For example, a β-d-galactosidase from *Paenibacillus thiaminolyticus* was found to have fucosidase activity [63]. Further experimental work will be needed to define the role of the multiple β-galactosidases reported here in dairy related *Paenibacillus* spp. isolates. For example, the different β-galactosidases may be expressed under different conditions (e.g. different temperatures) or may facilitate use of diverse oligo- and polysaccharides in addition to lactose. In addition, our data suggest that *Paenibacillus* may represent a potential source of GRAS β-galactosidases for industrial applications.

### Comparison of the two *Paenibacillus* strains in clade I revealed cold-growth related genomic features only in the strain able to grow at 6°C in skim milk broth

Strains FSL H7-689 and FSL R5-192 are closely related strains of *Paenibacillus*, which both have *rpoB* allelic type 23 (Table 1) [5]. Accordingly, these two strains clustered in the same clade in the phylogenetic analysis (Figure 1) and presented a high genome-wide average nucleotide identity (97.39%; see Additional file 3). However, these strains differed in their abilities to grow at 6°C in skim milk broth (monitored over 21 days); while FSL H7-689 was able to grow under these conditions, FSL R5-192 was not (Table 2). We thus performed a comparative analysis of the genomes for these two strains to identify potential genomic features that may contribute to these distinct phenotypes. This analysis identified 216 and 479 functionally annotated genes unique to strains FSL H7-689 and FSL R5-192, respectively (Additional file 9). Genes only found in FSL H7-689 encode proteins involved in a number of functions including, for example, synthesis of non-ribosomal peptide antibiotics (NRPA), transmembrane transport (e.g. Fe^3+^ transport, drug exporters, sugar transport systems), antibiotic resistance (e.g. an aminoglycoside 3’-phosphotransferase), as well as mobile elements (e.g. transposases and phage related proteins) and a toxin-antitoxin system (see Additional file 9). While genes only found in FSL R5-192 encode many proteins involved in similar functions (e.g. NRPA synthesis, transmembrane transport, antibiotic resistance), more mobile elements were found only in this strain. For example, we identified two putative prophages that appear complete, and multiple transposons in FSL R5-192 (Additional file 9), while no complete prophages and only two transposons were identified in FSL H7-689. Importantly, prophages in FSL R5-192 do not seem to be interrupting genes related with cold growth. Different stressful conditions (e.g. acidity, osmolarity, high temperature [64,65]), could induce the prophage lytic cycle, and consequently lyse the cells; experimental evidence will be necessary through to determine whether cold-growth could induce the lytic cycle in the prophage identified here, thus preventing cold growth.

Interestingly, we identified, in the genome of FSL H7-689, one cluster of genes annotated as encoding a DNA methylase, a restriction endonuclease, and a DEAD box helicase (Additional file 9); these genes were absent in FSL R5-192 with upstream and downstream genes located in a single contig, indicating that genes are not missing due to a sequence gap. DEAD box helicases are proteins that participate in a number of functions, including cold adaptation [66-68]. In *Bacillus subtilis*, deletion of DEAD box helicases was found to cause a cold-sensitive phenotype [66] and in *Listeria monocytogenes* deletion mutants of DEAD box helicases were unable to grow at 3°C [69]. Another DEAD box helicase (CshA) was found in both strains of clade I. While the presence of two DEAD box helicases only in the strain able to grow at 6°C in skim milk broth could be one of the genomic features that contributes to the cold growth phenotype of this strain, further experimental work is necessary to elucidate the role of this protein in cold adaptation of FSL H7-689. In addition, novel unrelated DEAD box helicases cannot be excluded as contributing to the ability of some *Paenibacillus* to grow at low temperatures.

### Comparison of *Paenibacillus* clade II and clade III genomes revealed a number of genes encoding cold adapted features and lactose utilization in clade II strains

Clade II strains present different phenotypic characteristics than clade III strains; specifically, only clade II strains were able to grow at 6°C in skim milk broth and showed proteolysis on skim milk agar (Table 2). Initial comparative genomic analyses identified 914 and 1,070 orthologs present in only clade II or clade III strains, respectively. To identify genomic features and genes that may be linked to the unique phenotypic characteristics of clade II strains (e.g. cold growth), we performed an enrichment analysis for RAST subcategories among clade II and III genomes. This analysis identified nine RAST subcategories that were overrepresented in the clade II genomes (Additional file 10 and Additional file 11); these subcategories were also significantly overrepresented in a comparisons of “cold adapted *Paenibacillus*” (i.e., clade II and clade I strain FSL H7-689) and “non-cold adapted *Paenibacillus*” (i.e., clade III and clade I strain FSL R5-192) (Additional file 10). While most of these subcategories are involved in energy acquisition or in bacterial growth; some of these subcategories (e.g. “protein and nucleoprotein secretion system, type IV”; “di- and oligo-saccharides”) encode proteins that could facilitate growth of clade II strains in milk. The RAST subcategory “di- and oligo-saccharides” includes genes encoding essential enzymes for lactose and galactose uptake and utilization (Additional file 10). These findings suggest that clade II strains contain unique genomic features that may facilitate their growth in milk and dairy production associated environments.

**Figure 3:**
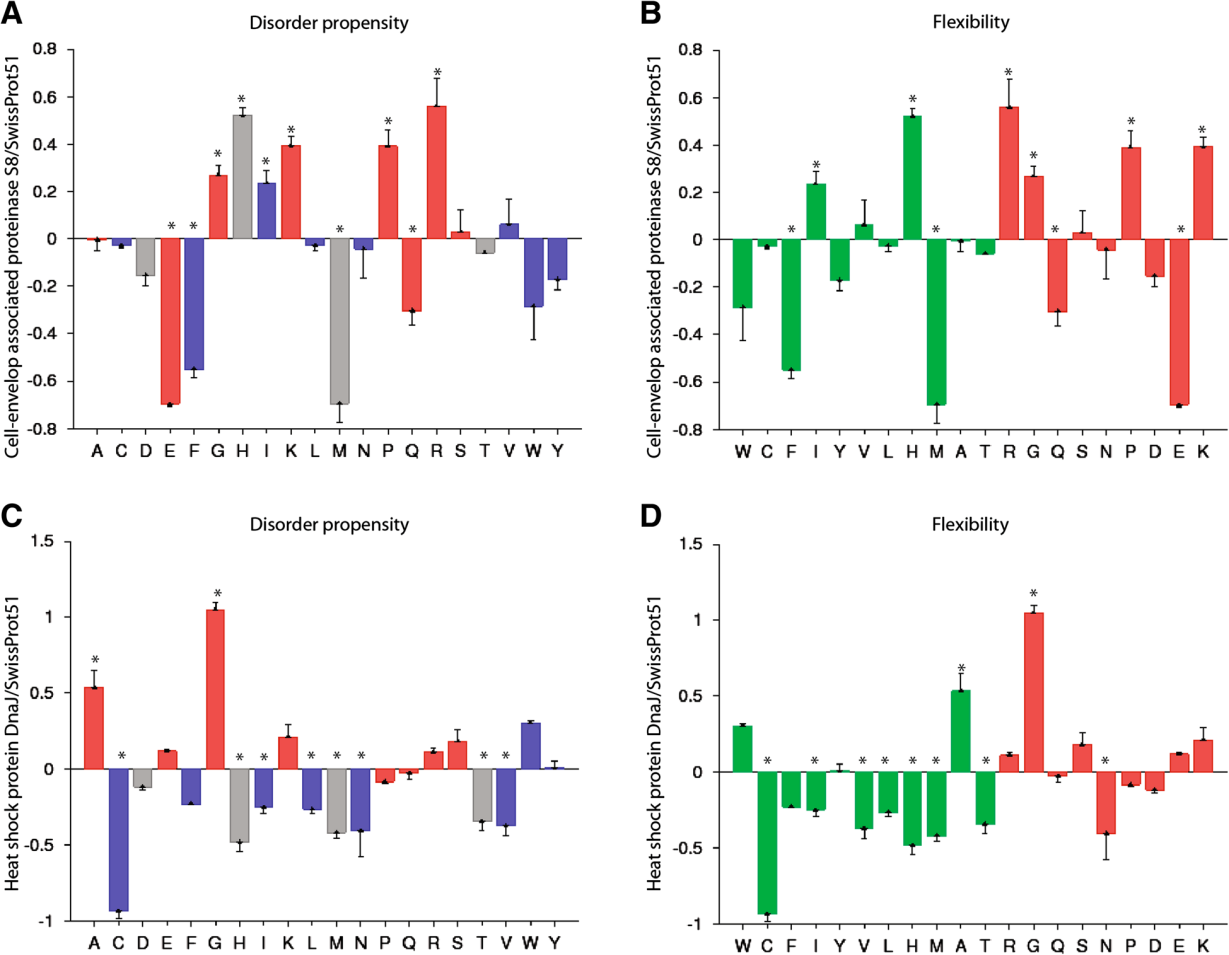
**Amino acid composition for DnaJ and cell-wall peptidase S8, the two proteins in clade II that showed cold-adapted features.** Analyses were performed using Composition Profiler [91] and the SwissProt 51 database [92]. Amino acids (aa) are shown on the x-axis. The y- axis represents the fractional difference between the aa distribution in the protein sample and in the background database; enriched aa are represented as positive values in the y-axis, depleted amino acids are represented as negative values in the y-axis. Bars with a *represent significant difference in aa composition (relative to the composition of the background proteins). Data for DnaJ are shown in panels **A** and **B**, while data for cell-wall peptidase S8 are shown in panel **C** and **D**. Panels **A** and **C** show aa composition features relevant to disorder propensity; aa residues are in alphabetical order and colored according to their disorder propensity: disorder-promoting residues are in red, order-promoting residues are in blue, and disorder–order neutral residues are in grey. Panel **B** and **D** show aa composition features relevant to flexibility; aa residues are ordered based on protein flexibility starting with the most rigid aa: rigidity promoting residues are in green and flexibility promoting residues are in red. Abbreviations: alanine (A), cysteine (C), aspartic acid (D), glutamic acid (E), phenylalanine (F), glycine (G), histidine (H), isoleucine (I), lysine (K), leucine (L), methionine (M), asparagine (N), proline (P), glutamine (Q), arginine (R), serine (S), threonine (T), valine (V), tryptophan (W), tyrosine (Y).

We also identified a number of specific genes that were only identified in clade II strains and that encode for properties that could be involved in the ability of these strains to grow in milk at cold temperatures, including genes encoding β-galactosidases (included in the RAST subcategory “di- and oligo-saccharides”) as well as genes encoding (i) peptide transport systems and peptidases and (ii) cold-adaptation related proteins. While we already reported the presence of several proteinases and peptide transport systems in all *Paenibacillus* genomes (in a previous section), some of these proteins appear uniquely encoded in the genomes of clade II strains. For example, two operons for peptide transport (oligopeptide and dipeptide transport systems) as well as several peptidases (cell-wall associated proteinase S8 and oligopeptidase F) were identified specifically in clade II (Additional file 12). Interestingly, these enzymes are associated with the three steps of bacterial casein breakdown, which includes (i) hydrolysis of casein into oligo- and dipeptides by cell envelope proteinases, (ii) peptide-transport-system-facilitated import of these peptides into the cell, and (iii) peptide degradation by intracellular peptidases [58,59]. We further analyzed the amino acid composition of the clade II specific proteins involved in casein hydrolysis to test the hypothesis that these proteins may contain specific structural features associated with cold-adaptation. In cold-adapted enzymes the protein structure is more flexible, which allows for catalysis at cold temperatures [70]; features associated with increased structural flexibility include disorder regions and amino acid bias (e.g. increase of glycine and decrease of acidic residues) [70-73]. Our analyses found that the cell-envelope associated peptidase S8 encoded by clade II strains (i) shows significant (*p* = 0.017) enrichment of disorder-promoting aa residues and (ii) shows enrichment of certain aa associated with cold adaptation (i.e., glycine and lysine) (Figure 3A, B). The peptidases of the S8 family have been found to function at extreme temperatures [74,75] and their stability is enhanced by calcium [74], important features for the growth in milk at refrigeration temperatures. In addition, genes encoding three β-galactosidases (β-gal types 10, 17, and 19) and several glycosyl hydrolases with possible β-galactosidase activity were identified in the genomes of clade II strains, but not in clade III strains (Additional file 12). We, however, did not identify cold-adapted features in any of these proteins. Interestingly, we found though that genes classified into glycosyl hydrolase families that have been shown to specifically have β-galactosidase activity (i.e., families 1, 2, 35 and 42) were over-represented in clade II (*p* = 0.044; overall Fisher’s exact test), suggesting that clade II may have unique carbohydrate metabolic capabilities (and specifically capabilities to hydrolyze lactose) that may facilitate growth in milk.

We also identified, in clade II strains, genes encoding several cold growth associated proteins that have been previously identified in other bacteria (Additional file 12), including (i) the low temperature requirement protein A (LtrA), which has been found to be important for growth at 4°C in *L. monocytogenes*[76]; (ii) chaperone DnaJ, which has been found to be up-regulated in *E. coli* and *Lactococcus* at cold temperatures [77,78], (iii) glutamate transport ATP-binding protein, which has been found to be up-regulated in *B. subtilis* at cold temperatures, and (iv) D-alanyl-D-alanine carboxypeptidase, which could decrease the cell wall resistance at cold temperature [70]. Among these proteins, DnaJ was identified to have cold-adapted features (flexibility and disorder promoting aa were both significantly enriched [*p* < 0.001]; see Figure 3C, D). Interestingly, two DnaJ-encoding genes were identified in clade II strains, one that is present in both the 3 clade II and the 2 clade III strains, and one that is present only in the 3 clade II strains and that encodes a DnaJ with cold-adapted features. In addition, in clade II strains we identified four aspartate aminotransferases, enzymes reported to be essential for growth of *Lactococcus lactis* in milk [79], as well as a number of proteins with no clear association with cold-adaptation (e.g. tellurite resistance operon, nitrogen fixation operon, and lactate dehydrogenase). Overall, we identified a number of proteins that could facilitate the growth of strains in clade II in milk at 6°C. Mapping of clade III genome sequencing reads against the clade II genome contigs that contained the clade II specific genes mentioned above (e.g. peptidase S8, β-galactosidases) confirmed that these genes were absent from the clade III genomes (see Additional file 12). To further understand the features associated with the cold tolerant phenotype of these strains; additional experimental data, including expression studies, are needed. The genes identified here do represent potential targets for these types of studies.

## Conclusions

Genomic analysis of selected sporeforming Bacillales isolated predominantly from milk allowed for identification of a number of genes encoding potentially relevant GRAS proteins such as potentially novel bacteriocins and NRPAs. Further comparative genomic analyses, focusing on *Paenibacillus* clades and strains that differed in their ability to grow in milk at refrigeration temperatures, identified a number of genomic features that likely allow some strains to grow at refrigeration temperatures and to break down milk associated proteins and carbohydrates at these temperatures. This study thus demonstrates how a genomic approach can be used to develop an improved understanding of microbial food spoilage, which will facilitate subsequent identification of genomic targets (e.g. β-galactosidases or peptidases only present in clade II) that can be used to control food spoilage or rapidly screen foods or raw materials for presence of bacteria with specific spoilage related genomic features.

## Methods

### Isolate selection

Isolates were selected for genome sequencing from a large collection of dairy-associated sporeforming Bacillales (>1,200) [5]. The ten isolates used here for genome sequencing (Table 1) were selected to (i) represent different genera of dairy associated sporeforming bacteria; (ii) represent various phenotypic and metabolic characteristics (Table 2) and (iii) represent the predominant *rpoB* allelic types associated with the dairy production system, using previously reported data [5]. We specifically selected the isolates to present strains that differ in their ability to (i) growth in skim milk broth at 6°C (monitored over 21 days), (ii) show proteolytic activity on skim milk agar, and (iii) show β-galactosidase hydrolysis on BHI X-gal. This approach was used to allow for comparative analyses to explore genomic features associated with the ability, of sporeforming Bacillales, to grow in milk at refrigeration temperatures.

### Illumina sequencing, assembly, and annotation

Genomic DNA from the selected isolates was prepared using phenol/chloroform DNA extraction [80]. Genomic DNA was quantified on the NanoDrop (NanoDrop products, Wilmington, DE) and standardized to 200 ng/ml. Isolate identity was confirmed via *rpoB* PCR prior to sequencing [5]. Genome sequencing was performed on the Illumina Hiseq 2000 at the Cornell University Life Sciences Core Laboratories Center (Ithaca, NY). Paired-end 2x100 bp reads were obtained and assembled *de novo* using Velvet and the VelvetOptimiser [81]. Assembled contigs were filtered to only include contigs >199 bp. Genome annotations were performed using a combination of RAST [82] and the NCBI Prokaryotic Genomes Automatic Annotation Pipeline (PGAAP) [83]. GenBank accession numbers are available in Table 1.

### Whole genome comparison and phylogenetic analysis

Previously sequenced closed genomes representing *Bacillus* spp. (n = 9) and *Paenibacillus* spp. (n = 6) were retrieved from NCBI (see Additional file 1) and used for comparative analyses. Genome alignments were performed with MAUVE [84], mapping alignments of homologous genes to a reference genome was conducted with GeneWiz 0.94 [85]. Phylogenetic analysis was performed using maximum likelihood methods implemented in RaxML version 7.0.4 [86] with 1,000 rapid bootstrap replicates, using amino acid sequences for 31 marker genes [36]. For this analysis, we selected 52 genomes representing sequenced Firmicutes (Additional file 1); genomes representing *Clostridium* spp. were selected to root the phylogenetic tree. Average nucleotide identity was calculated with JSpecies v1.2.1 [38].

### Phenotypic characterization

Isolates were stored at −80°C (15% glycerol, 85% BHI culture) and were grown on Brain-Heart Infusion (BHI) agar plates, which were incubated at 32°C for 24 h. β-galactosidase activity of isolates was assessed as previously described [5]. Briefly, isolates were streaked onto two BHI agar plates, one with and one without 100 μl of a 40 μg/ml solution of bromo-chloro-indolyl-galactopyranoside (X-Gal).

To assess cold growth, a single colony for each given isolate was first inoculated into BHI broth and grown for 24 h at 32°C. Cultures were diluted and inoculated into skim milk broth (BD, Franklin Lakes, NJ) to reach an initial inoculum of approx. 10^2^ to 10^3^ (plating showed that bacterial numbers ranged from 1.3-7.4 × 10^2^ CFU/ml). The inoculated skim milk broth was held at 6°C for 21 days and bacterial numbers were enumerated every 7 days by plating in appropriate dilutions on plate count agar (PCA; EMD Millipore, Swedesboro, NJ) using an Autoplate 4000 (Spiral Biotech, Norwood, MA). Bacterial colonies were counted after 24–48 h (depending on colony growth morphology) using Q-count.

### Antimicrobial resistance determination

The National Antimicrobial Resistant Monitoring System (NARMS) protocol for Gram positive bacteria was used to test for antimicrobial resistance. Minimal inhibitory concentrations (MIC) were determined using the Sensititre system (TREK Diagnostic Systems, Cleveland, OH), for the following 16 antimicrobials: chloramphenicol, ciprofloxacin, daptomycin, erythromycin, gentamycin, kanamycin, lincomycin, linezolid, nitrofurantoin, penicillin, streptomycin, synercid, tetracycline, tigecycline, tylosin, and vancomycin. The Clinical and Laboratory Standards Institute (CLSI) cut-offs, for bacterial isolates from animal origin, were used to interpret MIC values.

### Identification of proteins related to key phenotypes

Hidden Markov Models (HMM) for (i) relevant proteins (i.e., β-galactosidases, bacteriocins, glycosyl hydrolases) and (ii) proteins previously shown to be involved in relevant phenotypes (i.e., synthesis of non-ribosomal peptide antibiotics) were obtained from Pfam 26.0 protein families’ database [87] (see Additional file 13 for Pfam models used for the searches). Searches for protein sequence similarities were conducted with HMMER3 [88].We also conducted an additional search with the bacteriocin mining tool implemented in BAGEL3 [55]. Additional analyses were performed to identify, in the sequenced genomes, genes encoding proteolytic systems; briefly, proteins previously described to be involved in casein breakdown in lactic acid bacteria [59] were obtained from UniProt (http://www.uniprot.org/) and used as query sequences for BLAST searches against the 10 genomes sequenced. In addition, text searches of the genome annotations were performed to identify genes annotated as putative antimicrobial resistance genes.

### Ortholog comparisons

To identify genes that are unique to specific strains or clades (e.g. clades that only contain isolates with ability to grow at 6°C in skim milk broth), BLAST all against all searches were conducted with OrthoMCL v. 1.4 using the default settings [89]. For orthologs that were identified as unique to a given strain or clade, pBLAST searches against the GenBank database were performed to confirm the initial gene annotations. To confirm that genes classified as only present in clade II were absent from the clade III genomes, we also mapped the genome sequencing reads for the clade III genomes against clade II genome contigs that contained clade II specific genes. This read mapping was performed with SMALT version 0.7.5 [90]; using a word length of 17 and a sampling step size of 1.

### Analysis of amino acid composition

Amino acid sequences of selected proteins were analyzed for structural features related with cold-adapted enzymes [70]. Specifically, amino acids bias, disorder promoting regions and flexibility were analyzed using Composition Profiler [91] and the SwissProt 51 database [92] as a reference; 10,000 bootstrap iterations were performed.

### Enrichment analysis for RAST subcategories

Genes in all seven *Paenibacillus* were classified, by RAST, in 102 gene subcategories [82]. We assessed whether genes in each subcategory and genes identified by the glycosyl hydrolase HMMs were over-represented in a group of interest (e.g. clade II genomes) using the one-sided Fisher’s exact test implemented in R (version 2.13.0).

## Availability of supporting data

The sequence data supporting the results of this article are available on GenBank under the following accession numbers [GenBank: ASPZ00000000, ASPY00000000, ASQA00000000, ASPV00000000, ASPX00000000, ASPS00000000, ASPR00000000, ASPU00000000, ASPT00000000, ASPW00000000].

## Competing interests

The authors declare that they have no competing interests.

## Authors’ contributions

AA, MLR, NM, and RI: designed experiments; AA, NM and RI: conducted experiments; HdB and AA: participated in the sequence assembly; HdB, RHO, and AIMS: participated in genome analysis and interpretation; AIMS, MW, and KJB: wrote the manuscript. All authors read and approved the final manuscript.

## Supplementary Material

Additional file 1 **List of closed genomes used for phylogenetic analysis.** PDF file containing a table with the list of isolates representing *Bacillus*, *Paenibacillus,* and selected Firmicutes. Click here for file

Additional file 2 **Circular representation of BLAST comparisons for the *Paenibacillus* and *Bacillus* genomes.** A) BLAST comparison for all seven *Paenibacillus* using *P. lautus* Y4.12MC10 as reference genome. The inner circles in red and blue represent CDS in leading and lagging strands of *P. lautus* Y4.12MC10. In red from inside to outside are the genomes of FSL H8-457, FSL R5-808, FSL H8-237, in blue are the genomes of FSL R7-277 and FSL R7-269, and in green are the genomes of FSL H7-689 and FSL R5-192. B) BLAST comparison for the *Bacillus* and *Viridibacillus* genomes. The inner circles in red and blue represent CDS in leading and lagging strands of *B. weihenstephanensis* KBAB4. In red from inside to outside are the genomes of FSL H7-687, FSL R5-860, and FSL R5-213. Legends for BLAST identity percentages are found underneath for the respective figures. Click here for file

Additional file 3 **BLAST average nucleotide identity (ANIb), determined with the BLAST algorithm implemented in Jspecies, for *Paenibacillus* spp.** PDF file containing a table with the nucleotide identity values. Click here for file

Additional file 4 **List of putative antimicrobial and heavy metal resistance genes identified.** PDF file containing a table with the antimicrobial resistance genes identified in a given genome. Click here for file

Additional file 5 **Neighbor joining tree of the lincomycin resistant operon *lmrAB.* PDF file containing the tree.** In red are the strains that showed resistance. In parenthesis are the minimal inhibitory concentrations (MIC) determined for lincomycin. Click here for file

Additional file 6 **Number of putative casein breakdown associated proteins identified in the 10 genomes.** PDF file containing the number of proteins identified in the genomes sequenced here. Click here for file

Additional file 7 **Table containing the number of proteins representing putative β-galactosidases identified in the 10 genomes.** PDF file containing a table that details the numbers and types of β-galactosidases identified in the 10 genomes sequenced here. Click here for file

Additional file 8 **Heatmap of the distribution of β-galactosidases in the seven *Paenibacillus* genomes.** Blue indicates absence, green indicates presence, and dark green indicates two copies of a given β-galactosidase. Click here for file

Additional file 9 **Comparison of unique genes identified in FSL H7-689 (grows at 6°C) and FSL R5-192 (does not grow at 6°C).** PDF file containing a table that lists the proteins that were unique to either FSL H7-689 or FSL R5-192; hypothetical proteins are not included in the list. Click here for file

Additional file 10 **Subcategories over-represented in clade II and in cold-adapted strains.** PDF file containing a table that details the subcategories that are over-represented in clade II as compared to clade III, as well as in cold-adapted versus non-cold adapted strains. Click here for file

Additional file 11 **Number of genes in RAST subcategories shown to be significantly over-represented in clade II genomes as compared to clade III genomes.** For each RAST subcategory, gene numbers were color coded as representing (i) high number of genes in a specific subcategory (light gray) and (2) low number of genes in a given subcategory (dark gray); the cut-off between these categories were arbitrary. The two non cold-adapted clade III strains are shown above the three cold-adapted clade II strains. Click here for file

Additional file 12 **Clade II-specific genes that are putatively associated with the ability to grow in milk at refrigeration temperature.** PDF file containing clade II-specific genes. Click here for file

Additional file 13 **PFAM Models used in identification of genes encodings bacteriocins, non-ribosomal peptide antibiotics, β-galactosidases, and glycosyl hydrolases.** PDF file containing the accession numbers for the models used. Click here for file
