# Supplementary material for: Genomic comparison of sporeforming bacilli isolated from milk
Source: BMC Genomics. 2014 Jan 14;15:26. doi: 10.1186/1471-2164-15-26 (PMC3902026; doi:10.1186/1471-2164-15-26)
Supplement: Additional file 1 — List of closed genomes used for phylogenetic analysis. PDF file containing a table with the list of isolates representing Bacillus, Paenibacillus, and selected Firmicutes. [file 1471-2164-15-26-S1.pdf]

Additional file 1. List of closed genomes used for phylogenetic analysis

Strain	Species	GenBank acc. number	PMID of publication
E681	*Paenibacillus polymyxa*	CP000154	20851896
KNP414	*Paenibacillus mucilaginosus*	CP002869	22535950
HPL-003	*Paenibacillus terrae*	CP003107	22328761
JDR-2	*Paenibacillus* sp.	CP001656	22461553
Y4.12MC10	*Paenibacillus lautus*	CP001793	23408395
KBAB4	*Bacillus weihenstephanensis*	CP000903	17434157
ATCC 10987	*Bacillus cereus*	NC_003909	14960714
Ames	*Bacillus anthracis*	NC_003997	12721629
Al Hakam	*Bacillus thuringiensis*	NC_008600	17337577
FZB42	*Bacillus amyloliquefaciens*	NC_009725	17704766
168	*Bacillus subtilis*	AL009126	9384377
SAFR-032	*Bacillus pumilus*	CP000813	17895969
C-125	*Bacillus halodurans*	NC_002570	11058132
KSM-K16	*Bacillus clausii*	NC_006582	17429572
NG80-2	*Geobacillus thermodenitrificans*	NC_009328	17372208
DPC 4571	*Lactobacillus helveticus*	NC_010080	17993529
NCFM	*Lactobacillus acidophilus*	CP000033	15671160
ATCC 33323	*Lactobacillus gasseri*	NC_008530	17030793
NCC 533	*Lactobacillus johnsonii*	NC_005362	15006820
ATCC BAA-365	*Lactobacillus delbrueckii*	NC_008529	17030793
UCC118	*Lactobacillus salivarius*	NC_007929	16617113
V583	*Enterococcus faecalis*	NC_004668	19725962
WCFS1	*Lactobacillus plantarum*	AL935263	12566566
ATCC 25745	*Pediococcus pentosaceus*	NC_008525	17030793
PSU-1	*Oenococcus oeni*	NC_008528	17030793
ATCC 8293	*Leuconostoc mesenteroides*	CP000414	17030793
MGAS9429	*Streptococcus pyogenes*	NC_008021	16636287
A909	*Streptococcus agalactiae*	NC_007432	16172379
CNRZ1066	*Streptococcus thermophilus*	NC_006449	15543133
UA159	*Streptococcus mutans*	NC_004350	12397186
Challis	*Streptococcus gordonii*	CP000725	17720781
SK36	*Streptococcus sanguinis*	NC_009009	17277061
D39	*Streptococcus pneumoniae*	NC_008533	17041037
05ZYH33	*Streptococcus suis*	CP000407	17375201
SK11	*Lactococcus lactis*	NC_008527	17030793
QYMF	*Alkaliphilus metalliredigens*	NC_009633	unpublished
ATCC 27405	*Clostridium thermocellum*	NC_009012	unpublished
ATCC 33223	*Thermoanaerobacter pseudethanolicus*	NC_010321	unpublished
Hall	*Clostridium botulinum*	CP000727	18060065
E88	*Clostridium tetani*	NC_004557	12552129
ATCC 824	*Clostridium acetobutylicum*	NC_003030	11466286

13	*Clostridium perfringens*	NC_003366	11792842
630	*Clostridium difficile*	NC_009089	21349987
USA300_TCH1516	*Staphylococcus aureus*	NC_010079	17986343
JCSC1435	*Staphylococcus haemolyticus*	NC_007168	16237012
ATCC 12228	*Staphylococcus epidermidis*	NC_004461	12950922
ATCC 15305	*Staphylococcus saprophyticus*	NC_007350	16135568
Clip11262	*Listeria innocua*	NC_003212	11679669
SLCC5334	*Listeria welshimeri*	NC_008555	16936040
F2365	*Listeria monocytogenes*	NC_002973	15115801
EGD-e	*Listeria monocytogenes*	NC_003210	16428782
DSM 20601	*Listeria grayi*	NZ_ACCR02000000	unpublished
HTE831	*Oceanobacillus iheyensis*	NC_004193	12235376
Y4.12MC10	*Paenibacillus* sp.	CP001793	23408395
KBAB4	*Bacillus weihenstephanensis*	CP000903	17434157
