# Supplementary material for: Genomic comparison of sporeforming bacilli isolated from milk
Source: BMC Genomics. 2014 Jan 14;15:26. doi: 10.1186/1471-2164-15-26 (PMC3902026; doi:10.1186/1471-2164-15-26)
Supplement: Additional file 2 — Circular representation of BLAST comparisons for the Paenibacillus and Bacillus genomes. A) BLAST comparison for all seven Paenibacillus using P. lautus Y4.12MC10 as reference genome. The inner circles in red and blue represent CDS in leading and lagging strands of P. lautus Y4.12MC10. In red from inside to outside are the genomes of FSL H8-457, FSL R5-808, FSL H8-237, in blue are the genomes of FSL R7-277 and FSL R7-269, and in green are the genomes of FSL H7-689 and FSL R5-192. B) BLAST comparison for the Bacillus and Viridibacillus genomes. The inner circles in red and blue represent CDS in leading and lagging strands of B. weihenstephanensis KBAB4. In red from inside to outside are the genomes of FSL H7-687, FSL R5-860, and FSL R5-213. Legends for BLAST identity percentages are found underneath for the respective figures. [file 1471-2164-15-26-S2.pdf]

Additional file 2

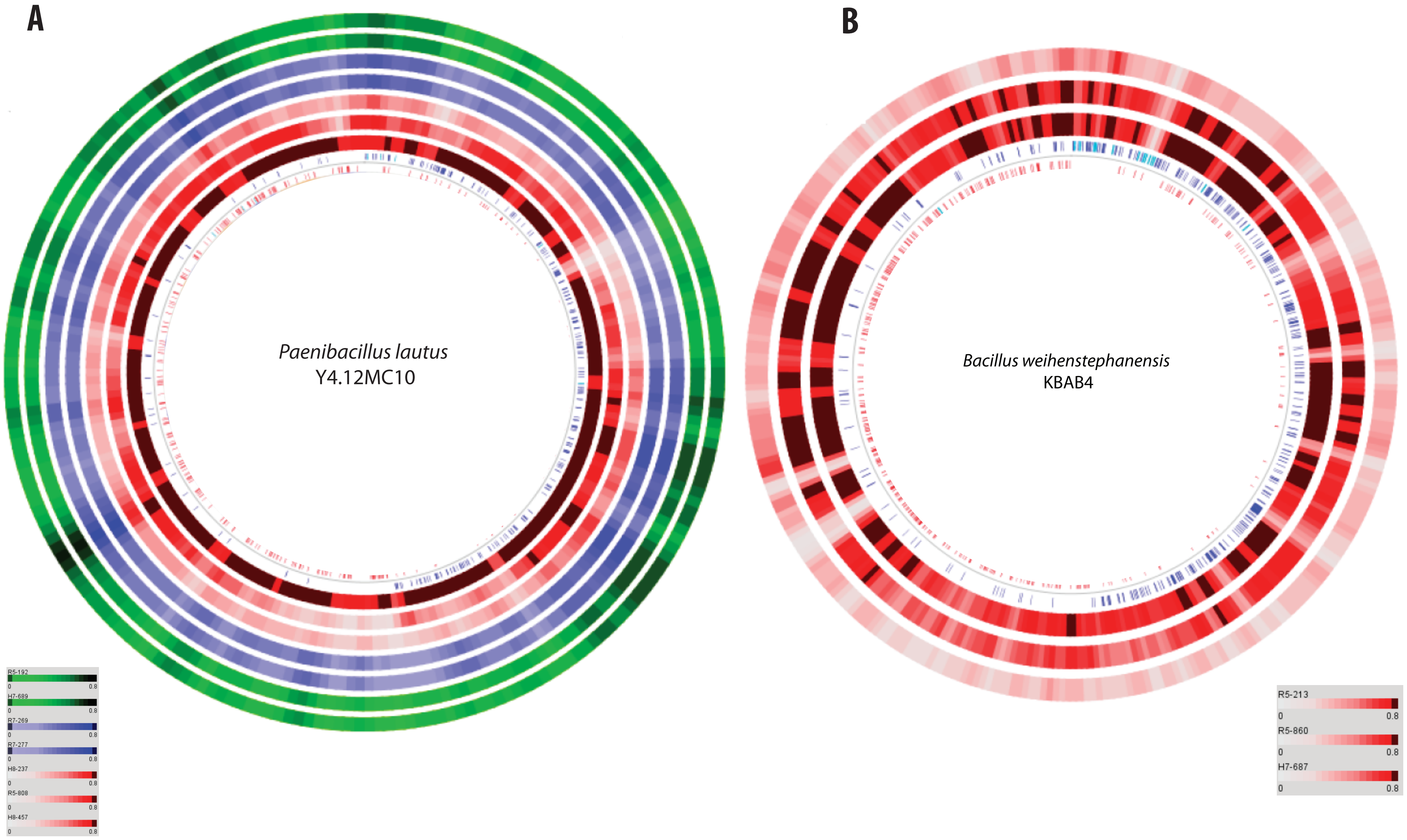

Circular representation of Blast comparisons for the Paenibacillus and Bacillus genomes. A) BLAST comparison for all *Paenibacillus* using *P. lautus* Y4.12MC10 as reference genome . The inner circles in red and blue represent CDS in leading and lagging strands of *P. lautus* Y4.12MC10. In red from inside to outside are the genomes of FSL H8-457, FSL R5-808, FSL H8-237, in blue are the genomes of FSL R7-277 and FSL R7-269, and in green are the genomes of FSL H7-689 and FSL R5-192. B) BLAST comparison for the *Bacillus* and *Viridibacillus* genomes. The inner circles in red and blue represent CDS in leading and lagging strands of *B. weihenstephanensis* KBAB4. In red from inside to outside are the genomes of FSL H7-687, FSL R5-860, and FSL R5-213. Blast identity percent are represented above for *Paenibacillus* and *Bacillus*. This figure was conducted by mapping proteins homology derived from WGS, determined by BLAST, against a reference genome as it is implemented in GeneWiz browser (http://www.cbs.dtu.dk/services/gwBrowser/).
