# Supplementary material for: Genomic comparison of sporeforming bacilli isolated from milk
Source: BMC Genomics. 2014 Jan 14;15:26. doi: 10.1186/1471-2164-15-26 (PMC3902026; doi:10.1186/1471-2164-15-26)
Supplement: Additional file 3 — BLAST average nucleotide identity (ANIb), determined with the BLAST algorithm implemented in Jspecies, for Paenibacillus spp. PDF file containing a table with the nucleotide identity values. [file 1471-2164-15-26-S3.pdf]

Additional file 3. BLAST average nucleotide identity (ANIb), determined with the BLAST algorithm implemented in Jspecies, for *Paenibacillus* spp.

Species	Strain (FSL)[2]	Percent of ANI (tetranucleotide signature)[1]						
		R7-269	R5-192	R7-277	H7-689	R5-808	H8-457	H8-237
P. sp.	R7-269	100 (100)	68.72 (0.82)	92.54 (0.99)	68.69 (0.81)	69.38 (0.88)	69.42 (0.85)	73.3 (0.85)
P. amylolyticus	R5-192		100 (100)	68.95 (0.82)	97.39 (0.99)	69.22 (0.81)	69.13 (0.72)	68.84 (0.84)
P. sp.	R7-277			100 (100)	68.89 (0.81)	69.42 (0.87)	69.95 (0.85)	73.57 (0.85)
P. amylolyticus	H7-689				100 (100)	69.01 (0.80)	69.14 (0.71)	68.99 (0.84)
P. glucanolyticus	R5-808					100 (100)	83.21 (0.95)	68.63 (0.87)
P. lautus	H8-457						100 (100)	68.54 (0.80)
P. odorifer	H8-237							100 (100)

[1] Strains with an ANI >95-96% and tetranucleotide value of >0.99 are likely to belong to the same bacterial species.
[2] The full strain designation includes the prefix FSL, e.g., FSL R7-269.
