# Supplementary material for: Genomic comparison of sporeforming bacilli isolated from milk
Source: BMC Genomics. 2014 Jan 14;15:26. doi: 10.1186/1471-2164-15-26 (PMC3902026; doi:10.1186/1471-2164-15-26)
Supplement: Additional file 4 — List of putative antimicrobial and heavy metal resistance genes identified. PDF file containing a table with the antimicrobial resistance genes identified in a given genome. [file 1471-2164-15-26-S4.pdf]

Additional file 4. List of putative antimicrobial and heavy metal resistance genes identified

		E-values (bit scores) of conserved domains [a,b]									
**Function**	**family**	**R5-860**	**H7-687**	**R5-213**	**H8-237**	**R7-277**	**R7-269**	**R5-192**	**H7-689**	**R5-808**	**H8-457**
β -lactam resistance	Metallo-β-lactamase (smart00849)	3.1e-36[b] (124.9)	4.79e-35[b] (121.8)	5.4e-25[b] (99.1)	5.6e-35[b] (124.2)	7.8e-32[b] (116.1)	1.6e-31[b] (113.0)	3.0e-30[b] (109.5)	1.3e-32[b] (91.4)	1.6e-33[b] (120.7)	2.3e-34[b] (123.0)
	Metal-dependent hydrolases of the β-lactamase (COG1234/ COG1235)	4.42e-50 (180.3)	8.89e-60[b] (178.3)	9.66e-61 (194.9)	2.30e-48 (163.0)	-	-	6.4e-48[b] (171.4)	3.7e-48[b] (171.8)	-	-
	β-lactamase (pfam00144)	2.2e-65[b] (203.1)	2.3e-62[b] (203.5)	9.5e-58[b] (195.4)	5.3e-64[b] (220.4)	5.7e-64[b] (215.0)	2.9e-61[b] (201.5)	3.1e-59[b] (205.6)	1.9e-60[b] (199.2)	4.1e-61[b] (198.4)	4.8e-59[b] (199.6)
	β-lactamase TEM (PRK15442)	2.95e-67 (210.4)	5.07e-69[b] (213.9)	-	-	-	-	-	-	-	-
	β-lactamase class A, PenP (COG2367)	-	-	-	-	-	-	-	-	1.61e-56 (184.5)	-
	β-lactamase class D (COG2602)	-	-	-	-	-	-	-	-	4.86e-80 (244.5)	4.78e-77 (236.8)
Aminoglycoside resistance	Aminoglycoside 3'-phosphotransferase (cd05155/cd05120)	5e-118[b] (364.3)	2.2e-28[b] (106.1)	-	-	3.7e-67[b] (207.4)	2.43e-33 (123.2)	5.3e-10[b] (35.4)	2.0e-10[b] (58.5)	6.1e-30 (112.0)	1.4e-52[b] (323.1)
	Aminoglycoside 3-N-acetyltransferase (pfam02522)	1.0e-110 (317.2)	4.6e-108 (310.7)	6.05e-86 (258.7)	1.3e-90[b] (270.2)	7.e-103[b] (297.6)	1.6e-46 (155.1)	8.1e-89[b] (261.8)	2.4e-87[b] (258.3)	3.5e-85[b] (252.5)	2.8e-93[b] (277.2)
	Streptomycin adenylyl transferase (pfam04439)	3.8e-104 (303.5)	8.4e-102 (297.8)	4.8e-113 (330.5)	-	-	-	-	-	-	-
Vancomycin resistance	VanW like protein (pfam04294)	7.5e-63[b] (193.4)	1.5e-63[b] (195.4)	-	9.5e-58[b] (188.5)	5.4e-61[b] (194.2)	3.7e-61[b] (194.6)	9.64e-58 (185.7)	1.06e-57 (185.7)	2.8e-68[b] (207.7)	5.6e-67[b] (208.1)
	VanZ like family (pfam04892)	1.53e-17 (73.5)	1.83e-04 (39.6)	8.93e-15 (67.3)	2.21e-14 (65.8)	-	-	-	-	-	-
	Glycopeptide resistance protein (COG4767)	-	-	-	-	-	5.22e-05 (42.3)	-	-	-	-
Chloramphenicol resistance	chloramphenicol resistance permease, RarD (PRK15430)	7.46e-67 (210.1)	9.69e-66 (207.0)	-	-	-	-	-	-	-	-
	chloramphenicol acetyltransferase, Cat (pfam00302)	-	6.5e-110 (312.3)	2.04e-94 (277.2)	4.0e-112 (322.3)	-	1.2e-109 (311.5)	5.9e-116 (327.7)	2.8e-116 (328.5)	1.7e-103 (296.1)	5.8e-107 (305.0)
	Chloramphenicol phosphotransferase (pfam07931)	-	-	-	3.00e-23 (92.1)	-	4.6e-26[b] (97.1)	7.31e-39 (97.1)	7.7e-38[b] (127.5)	-	-

Macrolide resistance	Macrolide transporter subunit, MacA (PRK11578)	-	-	-	-	-	-	-	6.81e-16 (76.1)	-	-
	Macrolide 2'-Phosphotransferase (cd05152)	1.82e-15 (65.7)	-	-	-	-	-	-	-	-	3.8e-133 (382.4)
Lincomycin resistance	Lincomycin resistance protein, LmrB	1.77e-77 (252.6)	1.18e-76 (250.7)	2.26e-79 (257.3)	8.56e-73 (240.3)	4.64e-75 (246.1)	9.04e-76 (248.0)	1.81e-65 (221.1)	9.15e-61 (208.3)	1.05e-59 (205.3)	3.33e-82 (265.0)
Bleomycin resistance	Bleomycin resistance protein (cd08349/cd07245)	3.72e-28 (99.5)	4.70e-36 (120.0)	-	-	3.05e-20 (84.9)	7.0e-11[b] (51.06)	1.95e-37 (121.9)	1.52e-25 (61.4)	2.69e-36 (118.8)	8.67e-13 (60.6)
	Glo_EDI_BRP_like_7 (cd08353/cd09012)	-	-	4.88e-56 (174.8)	3.57e-48 (153.1)	-	-	-	-	-	-
Fosfomycin resistance	fosfomycin resistance protein, FosB (cd08363)	7.74e-68 (199.6)	3.87e-66 (195.4)	1.33e-66 (200.8)	4.86e-24 (82.5)	-	1.54e-16 (71.0)	6.18e-58 (175.0)	5.62e-58 (175.0)	-	1.29e-60 (181.5)
Bacitracin and peptide antibiotics resistance	ABC_BcrA_bacitracin_resist (cd03268)	9.20e-90 (289.1)	9.33e-90 (291.0)	8.21e-88 (261.3)	4.49e-98 (289.4)	6.21e-96 (280.6)	6.6e-93[b] (293.7)	5.7e-86[b] (277.5)	3.0e-88[b] (284.4)	6.1e-96[b] (284.8)	3.0e-93[b] (278.7)
	lantibiotic protection ABC transporter permease (TIGR03733)	-	-	-	-	-	8.26e-64 (198.3)	5.30e-08 (37.6)	-	-	-
Bicyclomycin Resistance	bicyclomycin/multidrug efflux (PRK11102)	-	-	1.83e-65 (215.1)	2.23e-60 (202.0)	2.77e-66 (214.3)	6.13e-66 (216.7)	5.21e-66 (213.6)	-	3.9e-82[b] (255.6)	9.7e-70[b] (226.7)
Daunorubicin resistance	DrrA (cd03265)	4.4e-88[b] (274.6)	-	-	1.86e-62 (200.6)	-	-	-	-	-	-
Arsenical resistance	Arsenite efflux pump ACR3 (TIGR00832/pfam02040)	2e-154[b] (435.2)	6.85e-57 (216.3)	6.8e-135 (390.5)	5.9e-175 (497.7)	0e+00 (553.2)	6.46e-41 (140.7)	1.22e-55 (189.2)	7.00e-73 (189.2)	8.94e-37 (101.2)	0e+00 (556.3)
Multidrug resistance efflux pump	drug resistance transporter, EmrB/QacA (TIGR00711)	8e-112[b] (342.0)	9e-117[b] (354.7)	6.4e-91[b] (287.3)	5e-109[b] (337.0)	-	12e-102[b] (314.3)	5e-120[b] (359.7)	1e-119[b] (359.0)	-	1.4e-49[b] (176.8)
	Cation/multidrug efflux pump (COG0841)	4.e-178[b] (594.5)	0e+00 (602.6)	5e-174[b] (538.3)	1.2e-165 (515.6)	-	-	2.0e-158 (494.8)	8.7e-157 (494.4)	-	1.1e-168 (526.6)
	The Major Facilitator Superfamily (pfam07690)	7.8e-25[b] (87.3)	1.9e-25[b] (95.5)	1.4e-26[b] (107.5)	7.0e-20[b] (88.6)	3.3e-28[b] (112.9)	5.0e-21[b] (55.8)	1.3e-26[b] (112.9)	1.6e-26[b] (107.9)	41.e-33[b] (127.9)	3.0e-23[b] (99.0)
	Multidrug resistance protein (TIGR00880)	6.4e-21[b] (86.5)	4.18e-21 (86.9)	4.41e-23 (93.4)	1.57e-11 (61.1)	8.11e-17 (75.3)	-	-	1.44e-28 (71.5)	-	-
	drug resistance transporter, Bcr/CflA (TIGR00710)	7.6e-76[b] (238.8)	2.68e-77[b] (243.4)	1.43e-51 (178.7)	7.09e-51 (176.8)	-	-	-	-	-	-
	Na+-driven multidrug efflux pump (COG0534)	6.15e-70 (267.1)	5.85e-74[b] (297.1)	-	-	5.9e-75[b] (242.9)	1.2e-73[b] (276.8)	2.3e-77[b] (250.6)	3.3e-66[b] (253.3)	8.8e-83[b] (264.8)	4.9e-65[b] (259.0)
	drug efflux system protein MdtG (PRK09874)	8.5e-108 (322.2)	4.4e-106 (318.0)	-	-	-	5.00e-88 (271.4)	1.32e-61 (203.2)	2.0e-61[b] (202.4)		-
Hydroperoxide resistance	Peroxiredoxin (TIGR03561)	8.99e-76 (220.1)	1.99e-75 (219.3)	6.53e-60 (183.5)	1.8e-68 (204.3)	2.1e-69[b] (203.9)	6.8e-69[b] (202.7)	5.0e-68[b] (200.4)	5.07e-68 (200.4)	1.68e-61 (187.7)	2.22e-64 (191.2)

Cobalt-zinc-cadmium-copper resistance	heavy metal-translocating protein (TIGR01512)	1.e-152[b] (576.5)	-	-	-	0e+00 (537.6)	-	-	-	-	0e+00[b] (490.3)
	copper resistance, CutC (COG3142)	3.9e-52 (173.2)	-	-	-	3.92e-50 (162.4)	-	-	-	-	-
	Co/Zn/Cd efflux system (COG1230)	5.59e-87 (287.2)	7.63e-87[b] (286.9)	5.9e-104 (308.8)	1.3e-102 (120.8)	4.42e-88 (262.6)	4.77e-84 (259.5)	5.23e-84 (279.9)	4.58e-86 (285.7)	1.1e-86[b] (260.7)	5.41e-85 (259.9)
	Cu resistance protein, CopC (COG2372)	5.57e-25 (102.0)	-	-	-	-	-	-	-	5.46e-31 (90.9)	5.19e-30 (92.0)
	copper export protein, PcoD (COG1276)	-	2.84e-39 (150.2)	-	-	-	-	7.55e-31 (120.5)	8.17e-29 (120.5)	-	-
Aluminium resistance	Aluminium resistance protein (pfam06838)	0e+00 (759.9)	0e+00 (760.3)	0e+00 (668.6)	0e+00 (669.0)	-	0e+00 (658.6)	0e+00 (663.2)	0e+00 (664.4)	-	0e+00 (653.2)
Tellurite resistance	Tellurium resistance terD (cd06974)	9.6e-67[b] (200.6)	3.13e-67[b] (201.7)	4.73e-64 (197.9)	5.8e-43[b] (149.7)	1.0e-67[b] (202.9)	1.0e-49[b] (157.4)	5.4e-61[b] (185.9)	5.4e-61[b] (185.9)	-	-
	Toxic anion resistance protein (pfam05816)	1.1e-111 (327.6)	3.6e-111[b] (326.4)	1e-131[b] (384.2)	-	6.75e-21 (90.7)	-	3.09e-20 (88.7)	3.09e-20 (88.7)	-	-
Quaternary ammonium resistance	Membrane transporters of cationic drugs, EmrE (COG2076)	1.1e-27[b] (98.3)	5.7e-24[b] (97.9)	2.9e-34[b] (116.0)	1.4e-24[b] (89.5)	9.9e-28[b] (96.06)	5.7e-11[b] (59.08)	1.4e-18[b] (72.9)	9.6e-23[b] (92.6)	1.8e-30[b] (103.7)	8.2e-23[b] (86.4)

[a] e-values and bit scores were obtained with protein BLAST at NCBI
http://blast.ncbi.nlm.nih.gov/Blast.cgi?PROGRAM=blastp&BLAST_PROGRAMS=blastp&PAGE_TYPE=BlastSearch&SHOW_DEFAULTS=on&LINK_LOC=blasthome

[b] where multiple proteins were identified, the best hit was included in the table.
