# Supplementary material for: Genomic comparison of sporeforming bacilli isolated from milk
Source: BMC Genomics. 2014 Jan 14;15:26. doi: 10.1186/1471-2164-15-26 (PMC3902026; doi:10.1186/1471-2164-15-26)
Supplement: Additional file 5 — Neighbor joining tree of the lincomycin resistant operon lmrAB. PDF file containing the tree. In red are the strains that showed resistance. In parenthesis are the minimal inhibitory concentrations (MIC) determined for lincomycin. [file 1471-2164-15-26-S5.pdf]

Additional file 5

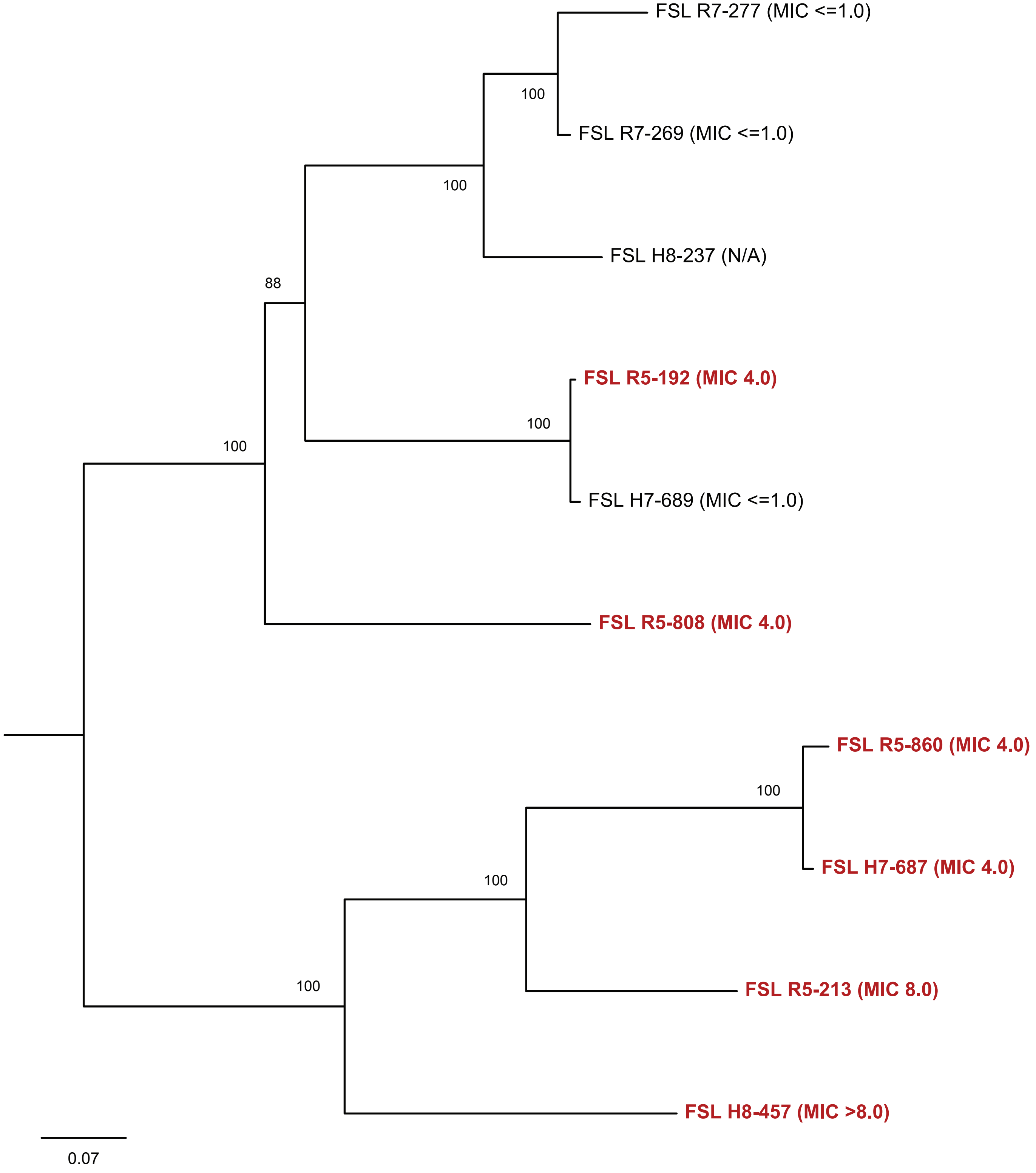

Neighbor joining tree of the lincomycin resistant operon LmrAB. Amino acid sequences were used for this tree**.** In red are the strains that showed resistance. In parenthesis are the minimal inhibitory concentrations (MIC) as determined for lincomycin.
