# Supplementary material for: Genomic comparison of sporeforming bacilli isolated from milk
Source: BMC Genomics. 2014 Jan 14;15:26. doi: 10.1186/1471-2164-15-26 (PMC3902026; doi:10.1186/1471-2164-15-26)
Supplement: Additional file 6 — Number of putative casein breakdown associated proteins identified in the 10 genomes. PDF file containing the number of proteins identified in the genomes sequenced here. [file 1471-2164-15-26-S6.pdf]

Additional file 6. Number of putative casein breakdown proteins identified in the ten genomes

		Number of homologous found in[1]									
Function	Proteins	R5-192	R7-269	H7-689	H8-237	R7-277	H7-687	R5-860	H8-457	R5-808	R5-213
Proteinase activity		+	+	+	+	+	+	+	-	-	-
Cell-wall bound proteinase	Prt	3	4	4	6	5	6	8	5	5	6
Peptide transporters	OppA DppA	9	7	7	9	8	14	14	9	6	14
	OppB DppB	9	7	8	8	7	7	7	9	7	14
	OppC DppC	9	7	8	7	7	6	7	9	8	14
	OppD DppD	9	5	5	8	6	4	4	9	7	14
	OppF DppF	9	5	4	7	6	4	4	9	7	14
	DtpT	0	0	0	1	0	3	2	0	0	1
Peptidases	PepP/PepQ	3	2	3	2	3	4	4	2	2	5
	PepA	1	2	2	2	2	3	3	2	2	3
	PepT	1	2	2	2	2	2	2	2	2	3
	PepF	4	4	3	4	4	6	7	4	4	6
	PepM	4	4	4	5	4	4	3	4	4	2
	PCP	2	0	1	0	0	1	1	1	1	1
	PepV	0	0	0	1	0	1	1	0	1	1
	PepI/PepL/PepR	0	0	0	0	0	0	1	0	0	0
	PepN	1	1	2	1	1	0	0	1	2	0
	PepX	0	0	0	0	0	1	1	0	0	0
	PepD	0	0	0	0	0	0	0	0	0	0
	PepO	0	0	0	0	0	0	0	0	0	0
	PepE/PepC	0	0	0	0	0	0	0	0	0	0
Transcriptional regulator	CodY	0	0	0	0	0	1	1	0	0	1

[1] HMM models for the casein proteolytic system of Lactic acid bacteria were used to search for these proteins in the genomes sequenced here; predicted proteins were validated using the Blast algorithm.
