# Supplementary material for: Genomic comparison of sporeforming bacilli isolated from milk
Source: BMC Genomics. 2014 Jan 14;15:26. doi: 10.1186/1471-2164-15-26 (PMC3902026; doi:10.1186/1471-2164-15-26)
Supplement: Additional file 7 — Table containing the number of proteins representing putative β-galactosidases identified in the 10 genomes. PDF file containing a table that details the numbers and types of β-galactosidases identified in the 10 genomes sequenced here. [file 1471-2164-15-26-S7.pdf]

Additional file 7.Table containing the number of proteins representing putative β-galactosidases identified in the 10 genomes.

Strain (FSL)	Species	β-gal activity	No. of proteins representing putative β-galactosidases[1] (distribution of β-galactosidases orthologs)
R5-860	*Bacillus* sp.	-	0
H7-687	*Bacillus weihenstephanensis*	-	0
R5-213	*Viridibacillus arenosi*	-	0
H8-237	*Paenibacillus odorifer*	+	16 (2-6,8,10,11,14,17,19-23)
R7-277	*Paenibacillus* sp.	+	19 (1-7,10-11,13-17,19,21,29,31)
R7-269	*Paenibacillus* sp.	+	17 (1-5,7,10-19)
R5-192	*Paenibacillus amylolyticus*	+	13 (1-11,19)
H7-689	*Paenibacillus amylolyticus*	+	13 (1-11,19)
R5-808	*Paenibacillus glucanolyticus*	+	9 (2,4,5,8,11,14,16,20,22)
H8-457	*Paenibacillus lautus*	+	18 (2-5,8,11,14,16,22-27,28,30)

[1] 33 β-galactosidases were identified and named from 1 to 33.
