# Supplementary material for: Genomic comparison of sporeforming bacilli isolated from milk
Source: BMC Genomics. 2014 Jan 14;15:26. doi: 10.1186/1471-2164-15-26 (PMC3902026; doi:10.1186/1471-2164-15-26)
Supplement: Additional file 8 — Heatmap of the distribution of β-galactosidases in the seven Paenibacillus genomes. Blue indicates absence, green indicates presence, and dark green indicates two copies of a given β-galactosidase. [file 1471-2164-15-26-S8.pdf]

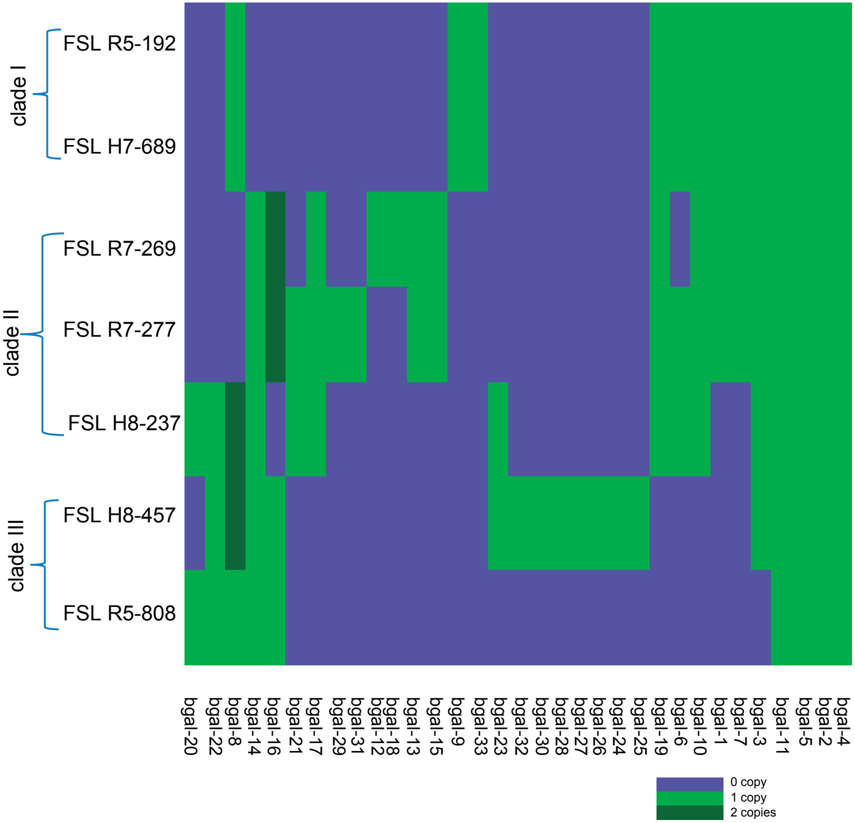

Additional file 8. Heatmap of the distribution of β-galactosidases in the seven *Paenibacillus* genomes. Blue indicates absence, green indicates presence, and dark green indicates two copies of a given β-galactosidase
