# Supplementary material for: Genomic comparison of sporeforming bacilli isolated from milk
Source: BMC Genomics. 2014 Jan 14;15:26. doi: 10.1186/1471-2164-15-26 (PMC3902026; doi:10.1186/1471-2164-15-26)
Supplement: Additional file 9 — Comparison of unique genes identified in FSL H7-689 (grows at 6°C) and FSL R5-192 (does not grow at 6°C). PDF file containing a table that lists the proteins that were unique to either FSL H7-689 or FSL R5-192; hypothetical proteins are not included in the list. [file 1471-2164-15-26-S9.pdf]

Additional file 9. Comparison of unique genes identified in FSL H7-689 (grows at 6°C) and FSL R5-192 (does not grow at 6°C).

Unique to FSL H7-689

**Gene function**[1]	**No. Orfs in FSL H7-689 (location)**
Non-ribosomal peptide antibiotic	15 (17,970-51,516)
Methyltransferase, protein kinase, NTPases	6 (299,004-308,123)
Fe3+ transport system	2 (43,876-46,789)
Platelet-activating factor	1 (178,435-179,940)
transposase	1 (63,518-64,636)
Proline-recognition domains	1 (82,975-83,739)
NlpC/p60-like peptidase & RNA polymerase sigma-54 factor	2 (5,261-7,928)
Reverse transcriptases	1 (47,266-51,117)
Aminoglycoside 3'-phosphotransferase	1 (77,065-77,955)
Glycosyl hydrolase family 5	1 (60,490-62,235)
Methyl viologen resistance	2 (108,033-119,517)
Drug exporter	2 (267,644-271,417)
Integrase	1 (439,985-440,923)
Bacterial glycosyl hydrolases	1 (458,693-459,769)
Sugar transport system	5 (471,797-478,386)
Bleomycin resistance, ferritin-like, among others	16 (82,249-93,854)
Putative phage proteins	5 (399,296-407,704)
Response regulator	2 (652,69-653,532)
S-layer domain	1 (659,489-656,769)
Restriction modification system & dead/box helicase	10 (700,395-716,031)
Putative phage protein	4 (152,848-156,660)
Sugar transport system	5 (118,640-126,140)
Integrase, protein kinase, among others	8 (141,186-146,950)
O-Methyltransferase involved in polyketide biosynthesis	1 (238,713-239,627)
Abortive phage infection	1 (310,566-311,462)
Phage protein, among others	5 (2,363-5,825)
Phage proteins	4 (232,876-237,621)
Sugar transport, among others	16 (378,102-395,719)
Germination protein	1 (430,518-430,913)
Kinase & membrane protein	2 (443,482-444,267)
Toxin-antitoxin	2 (86,284-86,687)
Putative phage proteins	11 (8,490-21,403)
Non-ribosomal peptide antibiotic	32 (108,142-146,215)
Beta-glucosidase	1 (188,506-188, 951)
Abortive phage infection, among others	3 (200,239-202,206)
Drug transporter, among other	4 (131,196-135,720)
NAD-dependent epimerase, among others	3 (203,833-206,021)
Amino acid transport and	6 (377,008-382-328)

metabolism & metal transporter	
DNA helicase	1 (64,441-67,245)
Efflux pump system	3 (76,449-78,944)
Outer membrane lipoprotein & transcriptional regulators	3 (177,029-180,322)
Metallophosphoesterase	3 (167,529-171,018)
Sugar transport and metabolism	12 (204,963-219-706)
Lipoprotein, among others[*]	9 (241,078-246,703)

Unique to FSL R5-192

**Gene function**[1]	**No. Orfs in FSL R5-192 (location)**
Flagella, integrase & salic acid metabolism	14 (68,274-82,864)
Copper amine oxidase	1 (104,888-106,114)
Phosphatidylinositol phosphate kinase	1 (350,924-351,721)
Transposase & IS	2 (363,262-364,442)
Periplasmic iron-binding protein	1 (78,687-80,666)
Biotin uptake, sugar transporters, among others	13 (91,815-105,967)
Phage related proteins	2 (168,400-169,235)
Non-ribosomal peptide antibiotic, among others	20 (186,330-228,843)
Transposases & Tetratricopeptide repeat	5 (114,620-120,235)
NADPH-dependent FMN reductase[*]	1 (180,345-180,899)
Short-chain alcohol dehydrogenases	2 (22, 102-23,611)
Thermoresistant glucokinase	2 (97,934-99,569)
Transposase[*]	1 (1-400)
Sugar transporter, chitinase, cellulose, among others	8 (29,737-41,900)
Phage tail fibre	1 (108,462-110,228)
Beta-lactamase & several hypothetical proteins	9 (55,076-69,957)
Zinc-dependent metalloprotease	2 (72,359-73,569)
Metallo-beta-lactamase	2 (92,516-93,903)
Streptomycin 3''-adenylyltransferase	1 (123,957-124,523)
Retron-type reverse transcriptase[*]	1 (110-1,402)
Phage related proteins	13 (600,025-612,017)
Endonuclease, Haloacid dehalogenase, among others	12 (809,321-820,059)
Di-peptide transport system	13 (941,383-956,383)
Rod shape-determining protein RodA	2 (1,055, 962-1,057,643)
Partitioning protein ParA, among several hypothetical proteins	6 (1,141,824-1, 147,731)
RNA polymerase sigma factor	2 (1,310,887-1,313,002)
Methyltransferase	1 (1,386,288-1,385,962)
Nucleoside deaminases	1 (1,439,807-1,440,352)
Acetyltransferase	1 (51,143-51,709)

Bleomycin resistance & Disulfide Oxidoreductases	4 (92,321-95,323)
hydroxymyristoyl-[acyl carrier protein] dehydratase and others	5 (110,559-114,738)
Beta-xylosidase & Beta-glucosidase	3 (130,242-136,429)
Methyltransferase & helicase	2(189,224-192,285)
Arsenical resistance operon	3 (224,940-227,093)
Transposase*	2 (337,517-338,494)
Transposase*	1 (249-1427)
Transposase	4 (104,264-106,592)
S-layer protein*	1 (57,613-61,209)
Macrolide 2'-phosphotransferase	1 (85,071-86,009)
NUDIX hydrolase	1 (111,777-112,394)
NAD-dependent epimerase/dehydratase	2 (120,076-121,581)
Fibronectin type III protein	2 (190,375-192,127)
Transcriptional regulator	2(16,303-17,142)
Transposase*	1 (97,771-98,107)
Transposase*	1 (1-963)
Phytoene dehydrogenase & sigma factor 70	2(31,175-33,395)
ADP-ribose pyrophosphatase, among others	4 (87,753-90,746)
Sugar transport system	9 (115,603-129,948)
Type VII secretion target	2 (284,474-286,173)
Phage related proteins*	1-7,950
Phosphatase/phosphohexomutase, among others	5 (53,596-57,247)
Lipid A permease, MDR protein, among others	25 (94,004-119,120)
Gene regulation proteins	4 (154,465-161,485)
FAD-dependent oxidoreductas	4 (33,865-36,947)
Iron-siderophore & transposases	5 (67,317-72,082)
Cobalt transport system	7 (88,075-93,775)
Nucleoside Triphosphate Hydrolases	3 (83,434-85,706)
Aminoglycoside phosphotransferase	1 (186,506-187,459)
Beta-glucosidase & mobile element associated proteins*	9 (1-11,008)
L-proline glycine betaine ABC transport system	5 (26,039-30,021)
Glycoside hydrolase, among others	16 (60,141-77,893)
Protease, endonuclease, peroxidase	11 (84,464-98,267)
Metal chaperone	3 (149,296-150,632)
Beta-lactamase, phage protein, among others	8(237,326-246,024)
Phage (complete)*	30 (357,722-383,535)
Phage (complete)*	61 (1-39,974)
Transposase*	4 (1-4,759)
reverse transcriptase, dextranase,	22 (26,243-53,964)

among others	
Acetyltransferase	2 (277,604-279,590)
Transposase*	1 (375,634-376,596)

[1] Hypothetical proteins are not included.

*Genes located where contigs end or start.
