# Supplementary material for: Genomic comparison of sporeforming bacilli isolated from milk
Source: BMC Genomics. 2014 Jan 14;15:26. doi: 10.1186/1471-2164-15-26 (PMC3902026; doi:10.1186/1471-2164-15-26)
Supplement: Additional file 10 — Subcategories over-represented in clade II and in cold-adapted strains. PDF file containing a table that details the subcategories that are over-represented in clade II as compared to clade III, as well as in cold-adapted versus non-cold adapted strains. [file 1471-2164-15-26-S10.pdf]

Additional file 10. Subcategories over-represented in clade II and in cold-adapted strains

RAST Subcategory	Clade II vs clade III *p*-value[1]	Cold-adapted vs non-cold-adapted *p*-value[1]	Example of proteins in a given subcategory	Potential implication for cold growth in milk
Inorganic sulfur assimilation	2.86e-11	9.68e-6	Sulfate binding protein, sulfate transport permease, sulfite reductase	Unclear, but volatile sulfur is found in UHT milk, sulfur is one of the minerals in milk
Protein and nucleoprotein secretion system, Type IV	4.54e-9	6.08e-10	Type IV pilus biogenesis (PilV, PilT, PilC, PilV, PilM)	Surface motility, biofilm formation
Di- and oligosaccharides	1.46e-10	1.42e-7	Sugar ABC transporters, β-galactosidase, galactoside ABC transport protein, α-glucosidase, PTS system, beta-glucoside	Lactose and galactose uptake and utilization. Involved in energy acquisition
Lacto-N-Biose I and Galacto-N-Biose Metabolic Pathway[2]	1.49e-5	0.002	UDP-glucose 4-epimerase, Lacto-N-biose phosphorylase	Involved in Leloir pathway of galactose metabolism. Lacto-N-Biose I and Galacto-N-Biose metabolic pathway involved in intestinal colonization by *Bifidobacterium*. May be involved in energy acquisition
Membrane Transport - no subcategory	0.0005	0.001	Cobalt transporters CbiMNQO	Unclear, cobalt is an essential cofactor of coenzyme B12
One-carbon Metabolism	0.003	0.01	Serine-pyruvate aminotransferase, formaldehyde lyase, Formate--tetrahydrofolate ligase	Unclear, may be involved in energy acquisition
Biotin	0.003	0.05	Biotin operon repressor, biotin-	Unclear, biotin is an essential co-

			protein ligase, biotin synthase	factor for carboxylase enzymes. May be necessary for high efficiency energy acquisition
Stress response	0.008	0.008	Serine phosphatase, phosphodiesterase	Unclear, involved in gene regulation
Nucleosides and Nucleotides - no subcategory	0.03	0.05	Ribonucleotide reductase, hydantoin racemase, Purine nucleoside phosphorylase	Unclear, involved in DNA metabolism and may be involved in growth

[1]Actual *p*-value are reported; a Bonferroni corrected adjusted *p*-value cutoff would be $p < 0.00049$; this is a highly conservative correction and we leave it to the reader to use the cutoff they deem appropriate.

[2] This subcategory was designated in RAST as “carbohydrates-no subcategory; role: Lacto-N-Biose I and Galacto-N-Biose Metabolic Pathway”
