# Supplementary material for: Genomic comparison of sporeforming bacilli isolated from milk
Source: BMC Genomics. 2014 Jan 14;15:26. doi: 10.1186/1471-2164-15-26 (PMC3902026; doi:10.1186/1471-2164-15-26)
Supplement: Additional file 11 — Number of genes in RAST subcategories shown to be significantly over-represented in clade II genomes as compared to clade III genomes. For each RAST subcategory, gene numbers were color coded as representing (i) high number of genes in a specific subcategory (light gray) and (2) low number of genes in a given subcategory (dark gray); the cut-off between these categories were arbitrary. The two non cold-adapted clade III strains are shown above the three cold-adapted clade II strains. [file 1471-2164-15-26-S11.pdf]

		Strain	Biotin	Stress response	Protein and nucleoprotein secretion system	Membrane transport	Nucleosides and Nucleotides	Lacto-N-Biose I and Galacto-N-Biose Metabolic Pathway1	Inorganic sulfur assimilation	Di- and oligosaccharides
non cold-adapted	clade III	FSL R5-808	11	8	0	5	10	0	0	81
		FSL H8-457	6	14	0	3	12	0	0	99
cold-adapted	clade II	FSL R7-277	26	19	11	14	18	7	15	169
		FSL H8-237	9	21	13	12	18	8	17	134
		FSL R7-269	19	21	14	14	18	7	16	161

Additional file 11. Number of genes in RAST subcategory shown to be significantly over-represented in clade II genomes as compared to clade III genomes. For each RAST subcategory, gene numbers were color coded as representing (i) high number of genes in a specific subcategory (light gray) and (2) low number of genes in a given subcategory (dark gray); the cut-off between these categories were arbitrary. The two non cold-adapted clade III strains are shown above the three cold-adapted clade II strains.
