# Supplementary material for: Genomic comparison of sporeforming bacilli isolated from milk
Source: BMC Genomics. 2014 Jan 14;15:26. doi: 10.1186/1471-2164-15-26 (PMC3902026; doi:10.1186/1471-2164-15-26)
Supplement: Additional file 12 — Clade II-specific genes that are putatively associated with the ability to grow in milk at refrigeration temperature. PDF file containing clade II-specific genes. [file 1471-2164-15-26-S12.pdf]

Additional file 12. Clade II-specific genes that are putatively associated with the ability to grow in milk at refrigeration temperature

Function	Protein designation[1]	Best domain hit[6]
Peptide transport	**ABC-type amino acid transport system**	COG0765 **
	**Oligopeptide ABC transporter, OppA**	cd00995 ***
	**Oligopeptide transport system permease, OppB**	PRK09471**
	**Oligopeptide transport system permease, OppC**	PRK15406 *
	**Oligopeptide ABC transporter, OppA**	cd08504 ***
	**Dipeptide-binding ABC transporter, DppA**	cd00995 ***
	**Dipeptide transport system permease, DppB**	COG0601 **
	**Dipeptide transport system permease, DppC**	COG1173**
	**Dipeptide transport ATP-binding, DppD**	cd03257**
	**Oligopeptide transport ATP-binding, OppF**	cd03257 **
Peptidases	**Methionine aminopeptidase**	cd01086 **
	**cell-wall associated proteinase S8**	cd07487 ***
	**Zinc-dependent peptidase**	pfam08367 **
	**Oligopeptidase F**	TIGR02290***
	Peptidase U32	pfam01136 *
Lactose utilization	**Beta-galactosidase (βgal 19)[2]**	pfam02449***
	**Beta-galactosidase (βgal 10)[2]**	COG3250 *
	**Beta-galactosidase (βgal 17)[2]**	COG1874 ***
	**Family 2 glycosyl hydrolase (C162_31939)[3]**	pfam13692 *
	Family 1 glycosyl hydrolase (C162_10951)[3]	cd03814 ***
	**Family 1 glycosyl hydrolase (C162_02152)[3]**	pfam00232 ***
	Family 1 glycosyl hydrolase (C162_27322)[3]	pfam00232***
	Family 1 glycosyl hydrolase (C162_32219)[3]	pfam00232***
	**Family 4 glycosyl hydrolase (C162_17712)[3]**	PRK15014***
	Family 43 glycosyl hydrolase (C162_28009)[3]	cd08993 **
	**Family 43 glycosyl hydrolase (C162_19289)[3]**	cd08983**
Cold growth associated	**Low temperature requirement protein A, LtrA**	pfam06772 **
Heat shock	**Chaperone DnaJ[4]**	TIGR02349 *
Amino acid metabolism	Aspartate aminotransferase (C162_00778)[5]	cd00609 *
	Aspartate aminotransferase (C162_04139)[5]	cd00609 **
	Aspartate aminotransferase (C162_00773)[5]	COG0075 **
	**Aspartate aminotransferase (C162_09661)**	cd00609 **
	Glutamate transport ATP-binding protein	cd03262 ***
Carboxypeptidase	D-alanyl-D-alanine carboxypeptidase	pfam13539 *
Lipid degradation	**Lipase**	COG0657 *
Homolactic acid fermentation	L-lactate dehydrogenase	cd05292 ***

[1] In proteins bolded, gene absence in clade III was identified by OrthoMCL analysis and confirmed by mapping the clade III sequencing reads against the appropriate clade II sequences (see Methods); in proteins that are not in bold the absence was identified by OrthoMCL analysis.

[2] β-galactosidases represented multiple distinct aa sequences and were thus designated as β-gal 1 to 33 (see Fig. 2 and additional file 7 for details).

[3] Family 1, 2, 4, and 43 glycosyl hydrolases have been found to show beta-galactosidase activity; family 1 and 43 glycosyl hydrolases are each listed multiple times as the clade II genomes evaluated carried multiple copies of these genes, which showed different best domain hits; the locus tags for FSL R7-269 are shown in parenthesis.

[4] While two copies of DnaJ are encoded in the clade II genomes, one was found in all *Paenibacillus* spp. genomes; the DnaJ listed here is unique to clade II and shows highest homology to *Streptomyces* DnaJ.

[5] Aspartate aminotransferases are listed multiple times as the clade II genomes evaluated carried multiple copies of these genes, which showed different best domain hits; the locus tags for FSL R7-269 are shown in parenthesis.

[6] Protein BLAST was used to identify the best domain hit; e-values for these hits are reported categorically as * (e-value between 1e-10 to 1e-49); ** (1e-50 to 1e-99); and *** (>1e-100); the e-values indicated represent the range for the lowest e-value found among the three clade II genomes.
