# Supplementary material for: Genomic comparison of sporeforming bacilli isolated from milk
Source: BMC Genomics. 2014 Jan 14;15:26. doi: 10.1186/1471-2164-15-26 (PMC3902026; doi:10.1186/1471-2164-15-26)
Supplement: Additional file 13 — PFAM Models used in identification of genes encodings bacteriocins, non-ribosomal peptide antibiotics, β-galactosidases, and glycosyl hydrolases. PDF file containing the accession numbers for the models used. [file 1471-2164-15-26-S13.pdf]

Additional file 13. PFAM Models used in identification of genes encodings bacteriocins, non-ribosomal peptide antibiotics, β-galactosidases, and glycosyl hydrolases.

**Name**	**description**	**Pfam accession**
**Bacteriocins**		
BacteriocIIc_cy	Bacteriocin class IIc cyclic gassericin A-like	PF12173
Bacteriocin_II	Class II bacteriocin	PF01721
Bacteriocin class IId	Bacteriocin class IId cyclical uberolysin-like	PF09221
Bacteriocin_IIi	Aureocin-like type II bacteriocin	PF11758
Cloacin	Colicin-like bacteriocin tRNase domain	PF03515
Colicin_immun	Bacterial self-protective colicin-like immunity	PF09204
Colicin-DNase	DNase/tRNase domain of colicin-like bacteriocin	PF12639
EntA_Immun	Enterocin A Immunity	PF08951
Lactococcin	Lactococcin-like family	PF04369
Lactococcin_972	Bacteriocin (Lactococcin_972)	PF09683
Bacteriocin_IIc	Bacteriocin class II with double-glycine leader peptide	PF10439
**Non ribosomal peptide antibiotics**		
KR	KR domain	PF08659
FAE1_CUT1_RppA	FAE1/Type III polyketide synthase-like protein	PF08392
Docking	Erythronolide synthase docking	PF08990
Ferritin-like	Ferritin-like	PF12902
McyA_C	Microcystin synthetase C terminal	PF12593
Erythro-docking	Erythronolide synthase, docking	PF09277
ketoacyl-synt	Beta-ketoacyl synthase, N-terminal domain	PF00109
Ketoacyl-synt_C	Beta-ketoacyl synthase, C-terminal domain	PF02801
Cyclase_polyket	Polyketide synthesis cyclase	PF04673
p450	Cytochrome P450	PF00067
Polyketide_cyc	Polyketide cyclase	PF03364
ABM	Antibiotic biosynthesis monooxygenase	PF03992
Polyketide_cyc2	Polyketide cyclase	PF10604
**B-galactosidase**		
Glyco_hydro_42	Beta-galactosidase	PF02449
Bgal_small_N	Beta galactosidase small chain	PF02929
Glyco_hydro_42M	Beta-galactosidase trimerisation domain	PF08532
Glyco_hydro_42C	Beta-galactosidase C-terminal domain	PF08533

BetaGal_dom2	Beta-galactosidase, domain 2	PF10435
BetaGal_dom3	Beta-galactosidase, domain 3	PF13363
BetaGal_dom4_5	Beta-galactosidase jelly roll domain	PF13364
**Glycosyl hydrolases**		
Glyco_hydro_42	Beta-galactosidase	PF02449.10
Glyco_hydro_35	Glycosyl hydrolases family 35	PF01301.14
Glyco_hydro_2	Glycosyl hydrolases family 2	PF00703.16
Glyco_hydro_1	Glycosyl hydrolase family 1	PF00232.13
